# H1-antihistamines as antischistosomal drugs: *in vitro* and *in vivo* studies

**DOI:** 10.1186/s13071-020-04140-z

**Published:** 2020-06-01

**Authors:** Rogério P. Xavier, Ana C. Mengarda, Marcos P. Silva, Daniel B. Roquini, Maria C. Salvadori, Fernanda S. Teixeira, Pedro L. Pinto, Thiago R. Morais, Leonardo L. G. Ferreira, Adriano D. Andricopulo, Josué de Moraes

**Affiliations:** 1 Núcleo de Pesquisa em Doenças Negligenciadas, Universidade Guarulhos, Guarulhos, SP Brazil; 2 Instituto de Física, Universidade de São Paulo, São Paulo, SP Brazil; 3 Núcleo de Enteroparasitas, Instituto Adolfo Lutz, São Paulo, SP Brazil; 4 Laboratório de Química Medicinal e Computacional, Instituto de Física de São Carlos, Universidade de São Paulo, São Paulo, SP Brazil

**Keywords:** Schistosomiasis, Antischistosomal, *Schistosoma*, Antihistamines, Drug repositioning

## Abstract

**Background:**

Schistosomiasis is a socioeconomically devastating parasitic infection afflicting hundreds of millions of people and animals worldwide. It is the most important helminth infection, and its treatment relies solely on the drug praziquantel. Oral H1-antihistamines are available worldwide, and these agents are among the most widely used of all medications in children and adults. Given the importance of the drug repositioning strategy, we evaluated the antischistosomal properties of the H1-antihistamine drugs commonly used in clinical practices.

**Methods:**

Twenty-one antihistamine drugs were initially screened against adult schistosomes *ex vivo*. Subsequently, we investigated the anthelmintic properties of these antihistamines in a murine model of schistosomiasis for both early and chronic *S. mansoni* infections at oral dosages of 400 mg/kg single dose or 100 mg/kg daily for five consecutive days. We also demonstrated and described the ability of three antihistamines to induce tegumental damage in schistosomes through the use of scanning electron microscopy.

**Results:**

From phenotypic screening, we found that desloratadine, rupatadine, promethazine, and cinnarizine kill adult *S. mansoni in vitro* at low concentrations (5–15 µM). These results were further supported by scanning electron microscopy analysis. In an animal model, rupatadine and cinnarizine revealed moderate worm burden reductions in mice harboring either early or chronic *S. mansoni* infection. Egg production, a key mechanism for both transmission and pathogenesis, was also markedly inhibited by rupatadine and cinnarizine, and a significant reduction in hepatomegaly and splenomegaly was recorded. Although less effective, desloratadine also revealed significant activity against the adult and juvenile parasites.

**Conclusions:**

Although the worm burden reductions achieved are all only moderate, comparatively, treatment with any of the three antihistamines is more effective in early infection than praziquantel. On the other hand, the clinical use of H1-antihistamines for the treatment of schistosomiasis is highly unlikely.
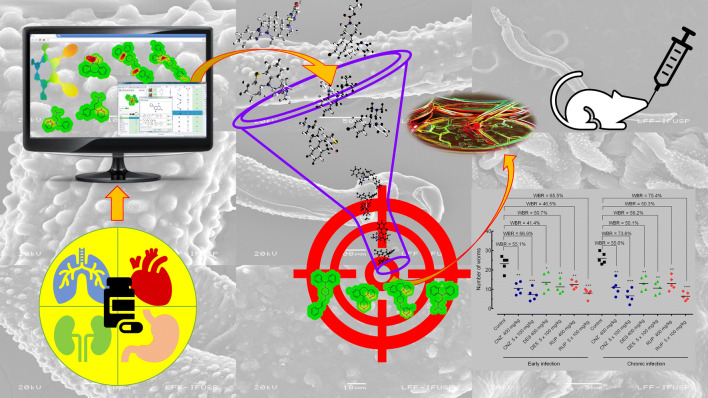

## Background

Infection with trematodes (blood flukes) of the genus *Schistosoma*, the causative agents responsible for schistosomiasis, causes chronic and debilitating disease in millions of people and animals worldwide [1]. Although not commonly fatal, schistosomiasis significantly contributes to a huge economic burden associated with low productivity and the perpetuation of the poverty cycle, as well as imposing a large burden on healthcare costs. Schistosomiasis is among the most prevalent parasitic diseases worldwide, and it is the most important human helminth infection in terms of global mortality and morbidity [2]. Approximately 800 million people may be at risk of infection worldwide, and almost 240 million are infected [3]. The combination of the global healthcare burden, the prevalence of these helminths, and limited treatment options have led to the inclusion of schistosomiasis on the World Health Organization’s list of neglected tropical diseases [4].

*Schistosoma mansoni* is the prevalent species in Africa, the Middle East, South America, and the Caribbean, in regions where the intermediate snail host, a freshwater snail of the genus *Biomphalaria*, is present. This parasite has a lifespan of several years and female schistosomes continuously produce eggs (each *S. mansoni* female worm can produce up to 300 eggs/day), which are able to pass through the intestinal lumen to be finally excreted with feces. Some of the eggs can be trapped in the tissues of the mammalian host instead of being excreted in the feces. Most, if not all, of the pathology in a schistosome infection results from the deposition of eggs in the tissues and the host’s response to them. The result is local, and systemic pathological effects include impaired cognition, anemia and growth stunting, as well as organ-specific effects, leading eventually to severe pathology such as hepatosplenomegaly and even death [5].

Praziquantel is a broadly effective trematocide and cestocide widely employed in veterinary and human medicine, and it is the only drug available to treat schistosomiasis. Praziquantel is very effective against adult worms (patent infection), but it is, unfortunately, poorly active against juvenile stages (prepatent infection), meaning that praziquantel must be given periodically for effective treatment and control [6]. In addition, widespread use of praziquantel in both humans and domestic animals [7, 8], along with the identification of laboratory and field isolates with reduced susceptibility to praziquantel [9, 10] raise serious concerns about the risk of selection of drug-resistant strains. Thus, new antischistosomal agents are needed, especially those targeting multiple stages of the parasite. For this reason, efforts to discover and develop novel antischistosomal agents have been intensified in recent years (for a review see [10, 11]). On the other hand, drug discovery is a lengthy and arduous process that inevitably struggles to deliver new therapies in a timely manner. Since the disease mainly affects poor people living in developing countries, pharmaceutical companies have little interest in developing new drugs. Thus, drug repurposing, the process of identifying new uses for existing drugs, is a promising strategy that has been used in recent years [12].

In schistosomes, and other flatworms, histamine is an important neuroactive substance [13] and G protein-coupled receptors (GPCRs) responsive to histamine have been described in *S. mansoni* [14, 15]. Due to their involvement in diverse biological and physiological processes, their pharmacological importance and potential as biological target, GPCRs are promising targets for new anthelmintic agents [16]. We have previously shown that promethazine, an old H1-antihistamine drug, had antischistosomal properties against *S. mansoni* adult worms *ex vivo* and in an animal model of schistosomiasis [17]. In view of these studies and in an attempt to explore drug repositioning strategy, here we evaluated the antiparasitic effect of a set of 21 H1-antihistamines commonly used in clinical practice. In this context, from phenotypic screening we found four antihistamine drugs that effectively killed *S. mansoni* adult worms *ex vivo*. Subsequently, these drugs were tested *in vivo* using an early and a chronic *S. mansoni* infection in a murine model. We also demonstrated the ability of these antihistamines to induce tegumental damage in adult worms through the use of scanning electron microscopy.

## Methods

### Drugs and reagents

All H1-antihistamines were purchased from Cayman Chemical (Ann Arbor, MI, USA), Sigma-Aldrich (St. Louis, MO, USA) and Toronto Research Chemicals (Toronto, Ontario, Canada). Praziquantel was kindly provided by Ecovet (São Paulo, SP, Brazil). The structures of all tested H1-antihistamines are shown in Additional file 1: Table S1.

RPMI 1640 culture medium, penicillin G/streptomycin sulfate, and inactivated fetal bovine serum (FBS) were purchased from Vitrocell (Campinas, SP, Brazil). HEPES buffer, glutaraldehyde solution, and dimethyl sulfoxide (DMSO) were purchased from Sigma-Aldrich.

### Maintenance of the *S*. *mansoni* life-cycle

The life-cycle of *S. mansoni* (BH strain) is maintained by passage through snails (*Biomphalaria glabrata*) and a mice (*Mus musculus*) as described by de Moraes [18]. The host snails were exposed to light (60 W incandescent light bulbs) for up to 3 h and subsequently cercariae of *S. mansoni* were harvested. Female Swiss mice, 3 weeks-old (purchased from Anilab, São Paulo, Brazil) were infected subcutaneously with approximately 150 cercariae. Both snails and mice were kept under environmentally controlled conditions (25 °C; humidity of 50%), with free access to water and food.

### *In vitro* anthelmintic assay

An *in vitro* anthelmintic assay was performed as previously described [19, 20]. Briefly, adult schistosomes (49 day-old) were collected from the portal system and mesenteric veins from infected mice (parasite *ex vivo*). Next, schistosomes were placed in RPMI 1640 culture medium supplemented with 10% FBS, containing 100 μg·ml^−1^ streptomycin 100 IU·ml^−1^ penicillin, and incubated in a 24-well culture plate (Corning, New York, NY, USA). Drugs were dissolved in DMSO to obtain stock solutions of 10 mM and then were tested at a concentration of 50 μM (one pair of parasites per well). Each drug was assessed in five replicates. Helminths were kept for 72 h (37 °C, 5% CO_2_) and their viability was monitored microscopically. The compounds that produced an effect superior to 90% after 72 h post-exposure underwent determination of their half maximum lethal concentration (LC_50_) using 1:2 serial dilutions from 0.78 to 50 µM [17, 21]. Each concentration was tested in triplicate, and experiments were repeated once. Negative control (using the highest concentration of DMSO) and positive control (praziquantel 2 µM) were included [22].

### Microscopy analysis

During the *in vitro* experiments, parasites were monitored using a light microscope (Leica Microsystems EZ4E, Wetzlar, Germany). In addition, schistosomes were visualized using a scanning electron microscope (JEOL SM-6460LV; JEOL Tokyo, Japan) whose experimental protocols were previously published [23]. Briefly, adult worms (control and treated groups) were fixed in 2.5% glutaraldehyde and mounted specimens were metalized with gold (Desk II sputter coater; Denton Vacuum LLC, Moorestown, NJ, USA) before observation under scanning electron microscopy.

### Studies in an animal model of schistosomiasis

Considering the *in vitro* results, we progressed cinnarizine, desloratadine and rupatadine to *in vivo* studies in both early and chronic *S. mansoni*-murine models as previously described [24]. Eighty Swiss mice, 3 weeks-old, were infected subcutaneously with 80 *S. mansoni* cercariae each. Animals were randomly divided into 16 groups (5 mice per group) and drugs were administered 21 days (early infection) or 42 days (chronic infection) post-infection by oral gavage. For treatment, drugs were dissolved in 2% ethanol in water (v/v) and tested at a single dose of 400 mg/kg or a dose of 100 mg/kg/day for five successive days. Groups of *S. mansoni*-infected control were given a corresponding amount of vehicle on the same timetable. At 56 days post-infection, all animals were euthanized by the CO_2_ method and dissected; parasites were then collected, sexed, and counted [25, 26]. Therapeutic activity was also based on the technique of qualitative and quantitative oograms in intestine, as well as the Kato-Katz method for quantitative fecal examination [27].

### Randomization and blinding

Animal studies are reported in compliance with the National Centre for the Replacement and Refinement & Reduction of Animals in Research (NC3Rs) ARRIVE guidelines. The mice were randomly assigned to the experimental groups, and pharmacological treatments were also performed randomly. The mice were euthanized in a random manner inside a group. All parameters (worm counts, measurement of the mass of the organs, quantitative and qualitative oogram, and quantitative fecal examination) were performed by different people (at least by two different investigators). Therefore, to eliminate bias in interpretation, manipulators of the experiments were not the same as the data analysts.

### Statistical analysis

Statistical analyses were performed using GraphPad Prism version 7 (GraphPad Software, San Diego, CA) in accordance with the recommendations in the pharmacology field [24]. All data from the *in vitro* anthelmintic experiments are presented as the mean ± standard deviation (SD) of at least three independent assays. LC_50_ values were calculated using sigmoid dose-response curves and 95% confidence intervals [28]. Kaplan-Meier survival analyses were also used to compare *in vitro* survival data, and *P*-values calculated using the log-rank (Mantel-Cox) test. For experimental analysis of animal studies, a parametric Dunnett’s test was applied to compare the control group with the treated group. The level of statistical significance was set to *P*   < 0.05 [17].

### Molecular and physicochemical properties

Molecular and physicochemical properties were calculated using the default parameters in the ADME/QSAR models of StarDrop version 6.6 (Optibrium, Cambridge, UK). The heat maps were performed using the same platform.

## Results

### *In vitro* efficacy of H1-antihistamine drugs against adult schistosomes

#### Effect of H1-antihistamines on parasite viability

**Fig. 1:**
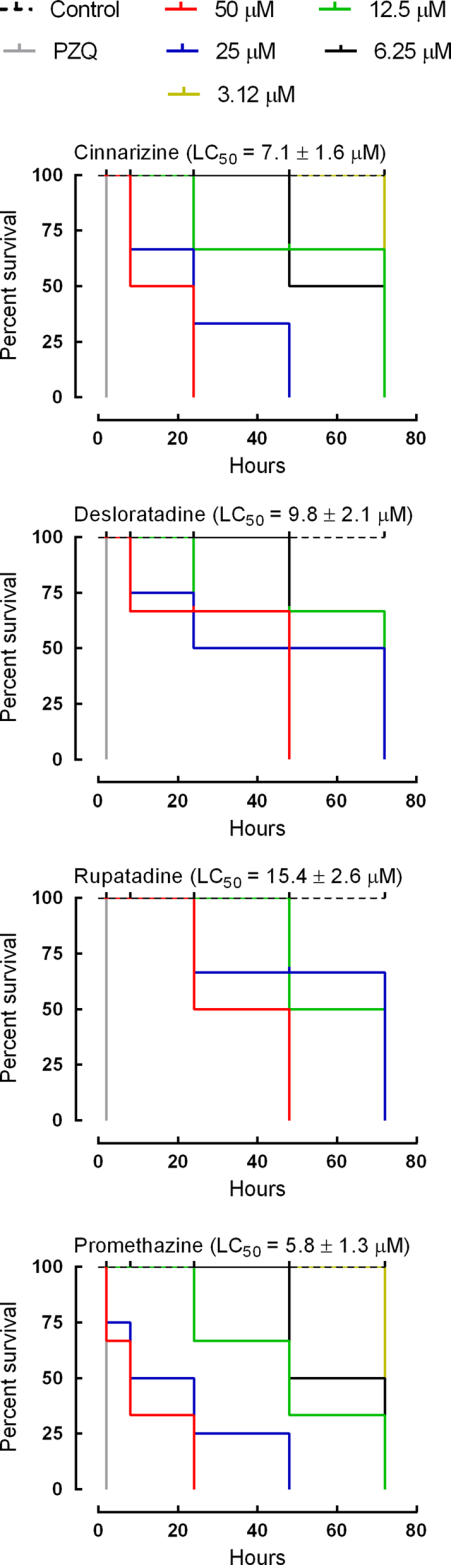
Viability of adult *S. mansoni* parasites *ex vivo* following exposure to H1-antihistamine drugs. Adult parasites were collected from the hepatic portal and mesenteric veins of mice and placed on plates containing the indicated concentrations of H1-antihistamines. Parasites were monitored for up to 72 h using a microscope and results are expressed as the percent mortality recorded by Kaplan-Meier survival curves. Mean values of viability were derived from a minimum of three experiments (*n* = 3), and each experiment was performed with five replicates. LC_50_ values were determined at 72 h. Control (dashed line): RPMI 1640 + 0.5% DMSO. PZQ, praziquantel at 2 μM

**Fig. 2:**
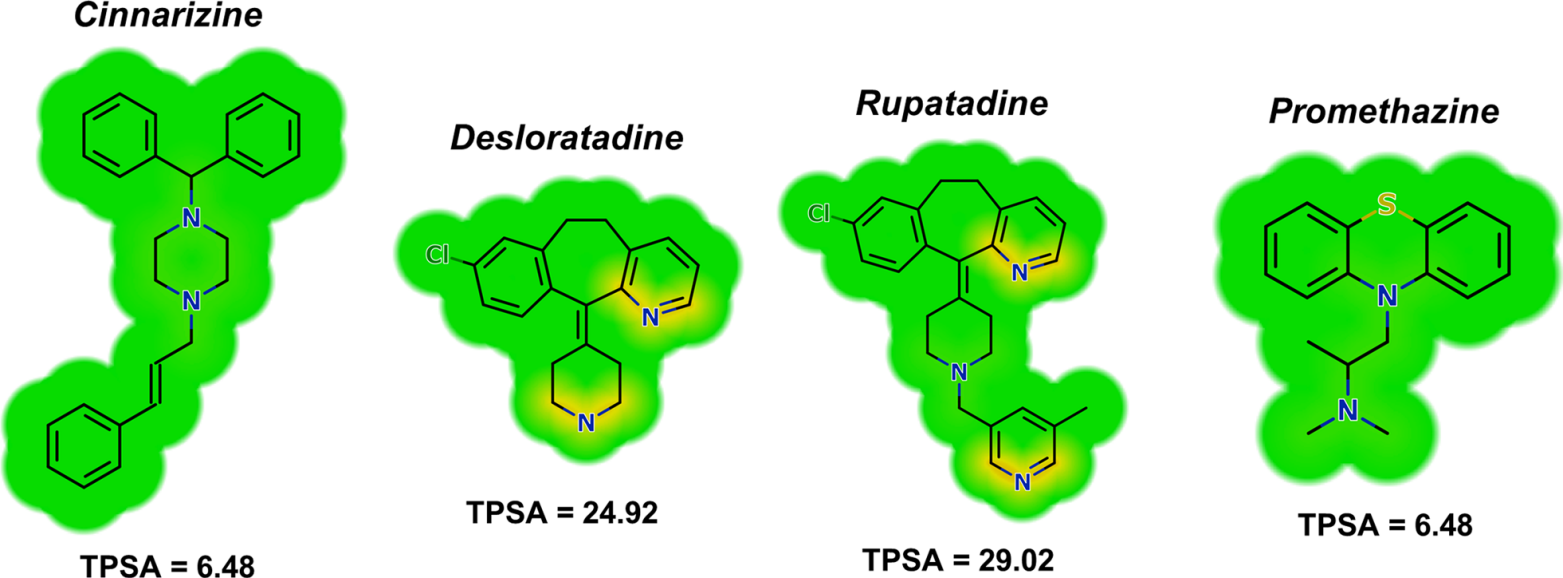
Heat maps for topological polar surface area (TPSA). The yellow regions contribute to increasing the value of TPSA, whereas the green regions have no influence

The 21 H1-antihistamine drugs were initially screened against adult schistosomes *ex vivo* at 50 µM. Of all H1-antihistamines tested, four (desloratadine, rupatadine, cinnarizine and promethazine) showed antischistosomal properties after 72 h, and these H1-antihistamines were further tested at a range of concentrations for their LC_50_ determination. Out of these, compared with the control group, cinnarizine, desloratadine, and promethazine achieved an LC_50_ below 10 μM, whereas rupatadine had a LC_50_ value of ~15 μM (Fig. 1). Comparison of LC_50_ values revealed that the order of potency was promethazine (Mantel-Cox signed-rank test: *χ*^2^ = 29.09, *df* = 6, *P*   < 0.0001), cinnarizine (Mantel-Cox signed-rank test: *χ*^2^ = 41.03, *df* = 6, *P*  < 0.0001), desloratadine (Mantel-Cox signed-rank test: *χ*^2^ = 28.68, *df* = 6, *P*  < 0.0001), rupatadine (Mantel-Cox signed-rank test: *χ*^2^ = 23.19, *df* = 5, *P*  < 0.001). The calculation of the molecular and physicochemical properties of the tested drugs (Additional file 1: Table S1) suggests that the antischistosomal activity may be correlated with the polar surface area of the compounds. The topological polar surface area (TPSA) values, which have been demonstrated to correlate well with passive transmembrane transport, were, in general, lower for the active drugs compared with the inactive compounds [29]. The active drugs had an average TPSA value of 15.70, while the inactive antihistaminic agents had the significantly higher TPSA value of 42.43. This indicates that the active drugs may be better diffused across parasite membranes. Heat maps constructed for the active compounds (Fig. 2) illustrate the contribution of the different portions of the molecules to TPSA.

**Fig. 3:**
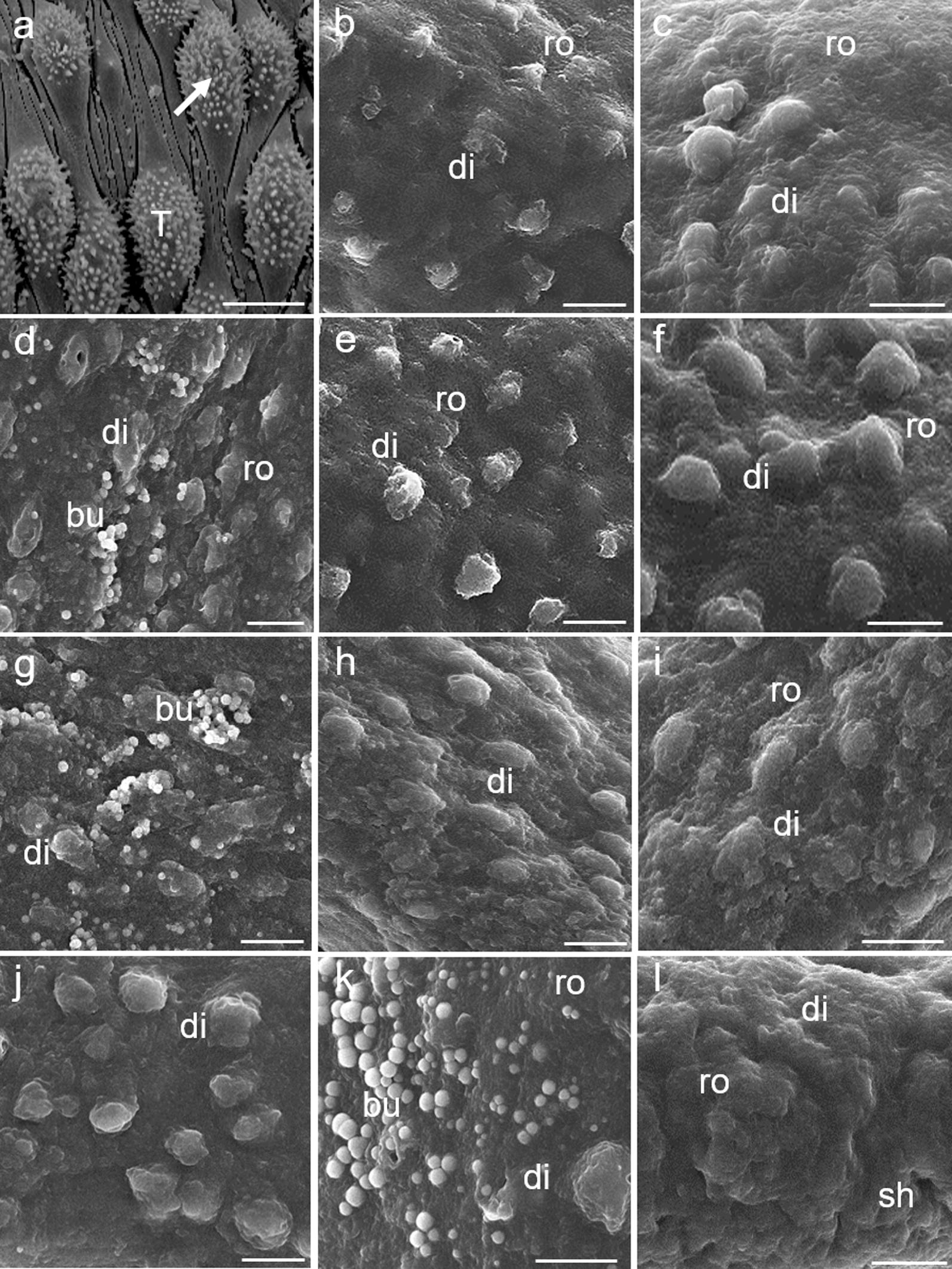
Scanning electron micrographs of *S. mansoni* after exposure to H1-antihistamines drugs. Adult parasites were collected from the hepatic portal and mesenteric veins of mice and placed on plates containing various concentrations of H1-antihistamines. Parasites were monitored at different times up to 72 h and micrographs of the mid-body region of male worms were obtained using a scanning electron microscope. **a** Control showing tubercles (T) and spines on the surface (arrow). **b**–**d** Twenty-four hours after incubation of cinnarizine 50 μM (**b**), desloratadine 50 μM (**c**) and rupatadine 50 μM (**d**). **e**–**g** Forty-eight hours after incubation of cinnarizine 25 μM (**e**), desloratadine 50 μM (**f**) and rupatadine 50 μM (**g**). **h**–**k** Seventy-two hours after incubation of cinnarizine 12.5 μM (**h**), desloratadine 25 μM (**i**), desloratadine 12.5 μM (**j**) and rupatadine 25 μM (**k**). **l** Praziquantel 2 μM. In figures **b**–**l**, the dorsal tegumental surface shows roughening (ro), disintegration (di), bubbles (bu) and shrinking (sh). Images were captured using a JEOL SM-6460LV scanning electron microscope. *Scale-bars*: 10 μm

The temporal effects of different concentrations of H1-antihistamine drugs on adult schistosomes are depicted in Fig. 3. Control parasites remained viable over the entire observation period of 72 h. Cinnarizine and promethazine (TPSA values of 6.48) were able to kill all schistosomes within 24 h of contact at a concentration of 50 μM. A slightly slower onset of action was observed when parasites were incubated with desloratadine or rupatadine (TPSA values of 24.92 and 29.02, respectively). This time-dependence is consistent with the TPSA values calculated for the active drugs. All adult schistosomes died within 48 h. In contrast, praziquantel had a very fast onset of action on schistosomes.

#### Effect of H1-antihistamines on parasite tegument

Since we have previously shown morphological changes in the tegument of the adult worms induced by promethazine [30], we conducted further studies with cinnarizine, desloratadine, and rupatadine using scanning electron microscopy. Figure 3a shows the tegument of a male parasite (control) depicting ridges and tubercles covered by spines that are somewhat uniformly distributed. By 24 h after incubation with 50 μM of cinnarizine (Fig. 3b), desloratadine (Fig. 3c), or rupatadine (Fig. 3d), extensive destruction was visible on the entire tegument of all adult worms analyzed. For example, rupture of the tegument along the whole dorsal body surface, including blebbing, shrinking and sloughing was visible. Moreover, tubercles had lost their spines. Similar morphological observations were made when the tegument of the schistosomes was evaluated after 48 h of exposure to cinnarizine 25 μM (Fig. 3e), desloratadine 50 μM (Fig. 3f) and rupatadine 50 μM (Fig. 3g). After incubation for 72 h, shrinking and swelling of the tegument was seen on all parasites exposed to cinnarizine 12.5 μM (Fig. 3h), as well as desloratadine at 25 μM (Fig. 3i) and 12.5 μM (Fig. 3j). Interestingly, massive bubbles were observed on all worms exposed to rupatadine at 25 μM after 72 h of incubation (Fig. 3k). The positive control (praziquantel 2 μM) caused massive shrinking and swelling of the tegument (Fig. 3l).

### The efficacy of H1-antihistamine drugs in mice harboring either early or chronic *S. mansoni* infection

Since promethazine was already tested *in vivo* and results published [17], we investigated the antischistosomal effect of cinnarizine, desloratadine, and rupatadine in mice harboring either early or chronic *S. mansoni* infection. Results were compared to the control infected but untreated animal harboring either early or chronic infection. Of note, all drugs were well tolerated, and all mice survived until the end of the experimental work.

#### Effect of H1-antihistamines on worm burden

**Fig. 4:**
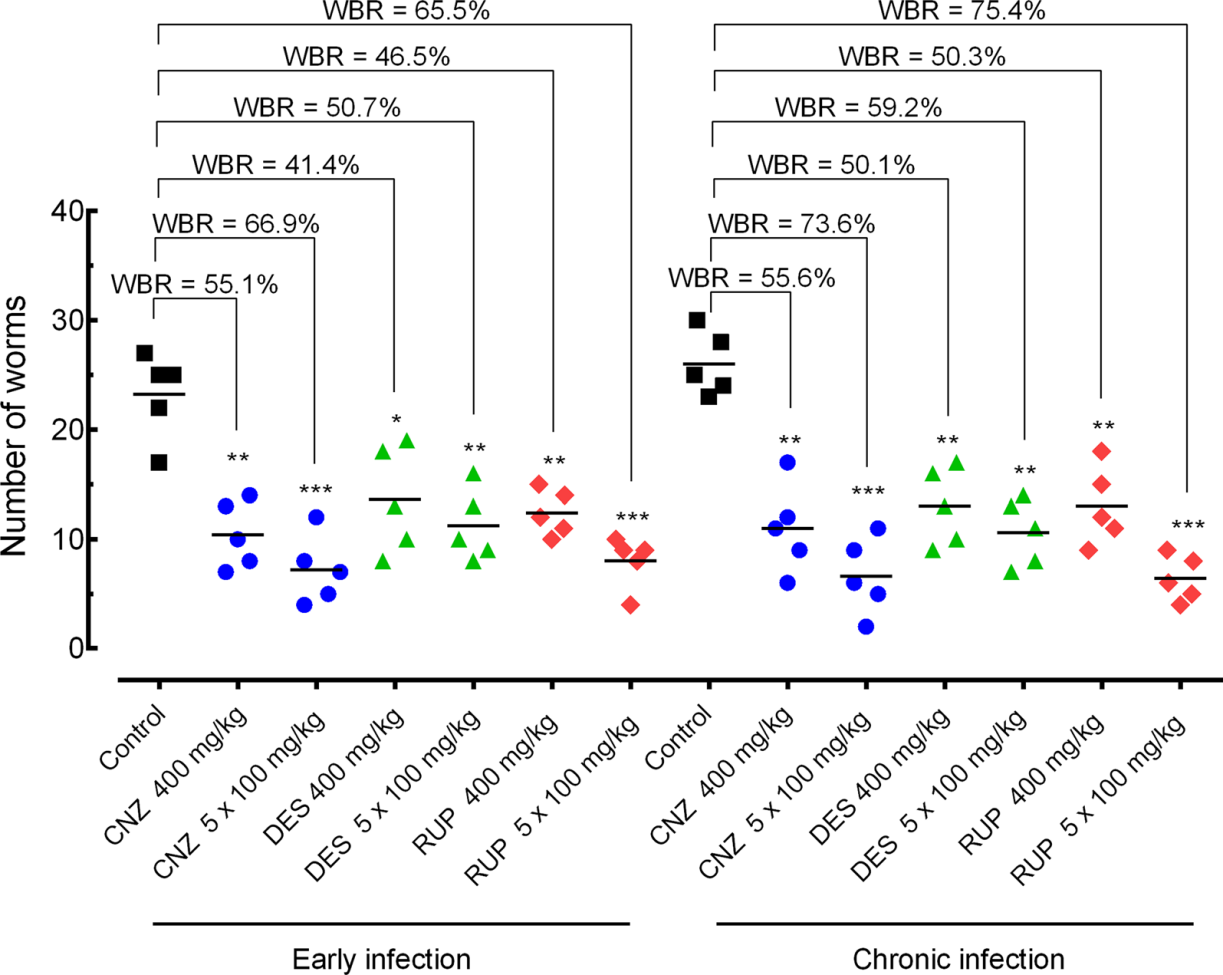
Effect of H1-antihistamine drugs on the parasite burden in a preclinical mouse model. Drugs were administered orally using a single dose of 400 mg/kg or 100 mg/kg for five consecutive days to mice harboring either early or chronic *S. mansoni* infection. On day 56 post-infection, all animals were humanely euthanized and parasite burdens were determined by sex (male and female parasites). Points represent data from individual mice (*n* = 5 per group). Horizontal bars represent median values. *P  < 0.05, **P  < 0.01, ***P   < 0.001 compared with infected untreated control by Dunnett’s test. *Abbreviations*: WBR, worm burden reduction; CNZ, cinnarizine; DES, desloratadine; RUP, rupatadine

Figure 4 summarizes the antischistosomal activity of cinnarizine, desloratadine, and rupatadine given in single or multiple oral doses in both early and chronic infection, compared to control *S. mansoni*-infected animals.

In early infection, using a single oral dose (400 mg/kg), cinnarizine achieved the highest worm burden reduction (55.1%; ANOVA: *F*_(13, 56)_ = 15.06, *P* = 0.0026). In the experiments where cinnarizine, rupatadine, and desloratadine were administered daily for 5 days (100 mg/kg), a decrease in total worm burden of 66.9% (ANOVA: *F*_(13, 56)_ = 15.06, *P* = 0.0003), 66.5% (ANOVA: *F*_(13, 56)_ = 15.06, *P* = 0.0004), and 50.7% (ANOVA: *F*_(13, 56)_ = 15.06, *P* = 0.0052), respectively, was observed.

In chronic infection, using a single oral dose (400 mg/kg), the H1-antihistamine drugs caused a total worm burden reduction ranging from 50.1% (ANOVA: *F*_(13, 56)_ = 15.06, *P* = 0.0059) to 55.6% (ANOVA: *F*_(13, 56)_ = 15.06, *P* = 0.0021). In the treatment using multiple oral doses (5 × 100 mg/kg), cinnarizine and rupatadine achieved high total worm burden reductions of 73.6% (ANOVA: *F*_(13, 56)_ = 15.06, *P*   < 0.0001) and 75.4% (ANOVA: *F*_(13, 56)_ = 15.06, *P* = 0.0052, *P*   < 0.0001), respectively. Lower but significant worm burden reduction values were obtained for desloratadine (59.2%; ANOVA: *F*_(13, 56)_ = 15.06, *P* = 0.0008).

#### Effect of H1-antihistamines on egg burden

The egg load was evaluated using the oogram technique (immature, mature and dead worms in the intestine) and the Kato-Katz technique for quantitative fecal examination.

**Fig. 5:**
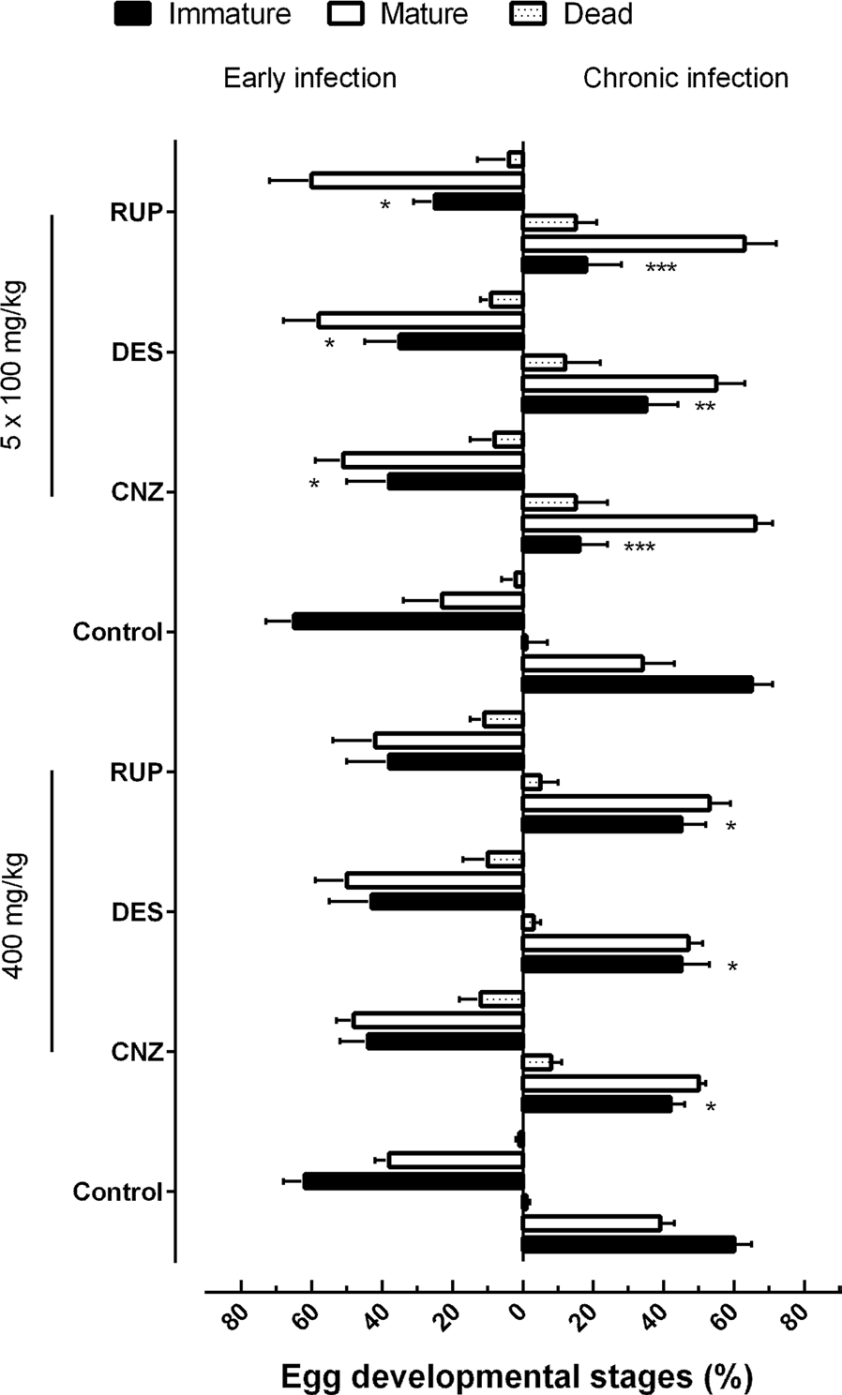
Effect of H1-antihistamine drugs on the egg developmental stage in a preclinical mouse model. Drugs were administered orally using a single dose of 400 mg/kg or 100 mg/kg for five consecutive days to mice harboring either early or chronic *S. mansoni* infection. On day 56 post-infection, all animals were humanely euthanized and egg burdens were determined by counting eggs in the intestine (quantitative and qualitative oogram technique). Data are presented as the mean ± SD (*n* = 5 per group). The numbers represent the percentages of egg reduction *vs* infected untreated control. *P   < 0.05, **P   < 0.01, ***P   < 0.001 compared with infected untreated control groups. *Abbreviations*: CNZ, cinnarizine; DES, desloratadine; RUP, rupatadine

Regarding the oogram, in early infection, multiple oral doses of any of the three antihistamines led to a significant reduction in the number of immature eggs (ANOVA: *F*_(7, 35)_ = 8.43, *P* = 0.026), whereas drugs administered in a single dose showed a non-significant reduction in the number of eggs when compared to control infected mice. In contrast, the number of immature eggs was highly reduced in mice harboring a chronic *S. mansoni* infection, especially when any of the three drugs were administered in multiple doses (ANOVA: *F*_(7, 35)_ = 8.43, *P* = 0.0006). The percentages of immature, mature and dead eggs are summarized in Fig. 5.

**Fig. 6:**
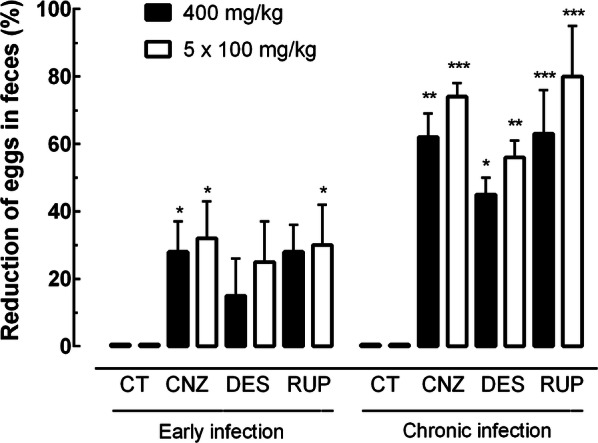
Effect of H1-antihistamine drugs on the egg burden in the feces in a preclinical mouse model. Drugs were administered orally using a single dose of 400 mg/kg or 100 mg/kg for five consecutive days to mice harboring either early or chronic *S. mansoni* infection. On day 56 post-infection, all animals were humanely euthanized and egg burden in feces was measured by Kato-Katz technique. Data are presented as the mean ± SD (*n* = 5 per group). The numbers represent the percentages of egg reduction *vs* infected untreated control. *P   < 0.05, **P   < 0.01, ***P   < 0.001 compared with infected untreated control groups by Dunnett’s test. *Abbreviations*: CNZ, cinnarizine; DES, desloratadine; RUP, rupatadine

With respect to fecal examination, cinnarizine and rupatadine administered daily for 5 days to mice harboring chronic infection greatly reduced the number of eggs in feces by 73.8% (ANOVA: *F*_(7, 35)_ = 8.43, *P*   < 0.0001) and 80.1% (ANOVA: *F*_(7, 35)_ = 8.43, *P*   < 0.0001), respectively. Under the same drug regimen, desloratadine showed moderate but significant reductions in egg burden (56.5%; ANOVA: *F*_(7, 35)_ = 8.43, *P*   < 0.0001). In the experiments where antihistamines were administered with a single dose (400 mg/kg), a lower percentage reduction in the number of eggs in fecal samples relative to control infected mice was observed, especially in animals with early infection (Fig. 6).

#### Effect of H1-antihistamines on hepato- and splenomegaly

**Fig. 7:**
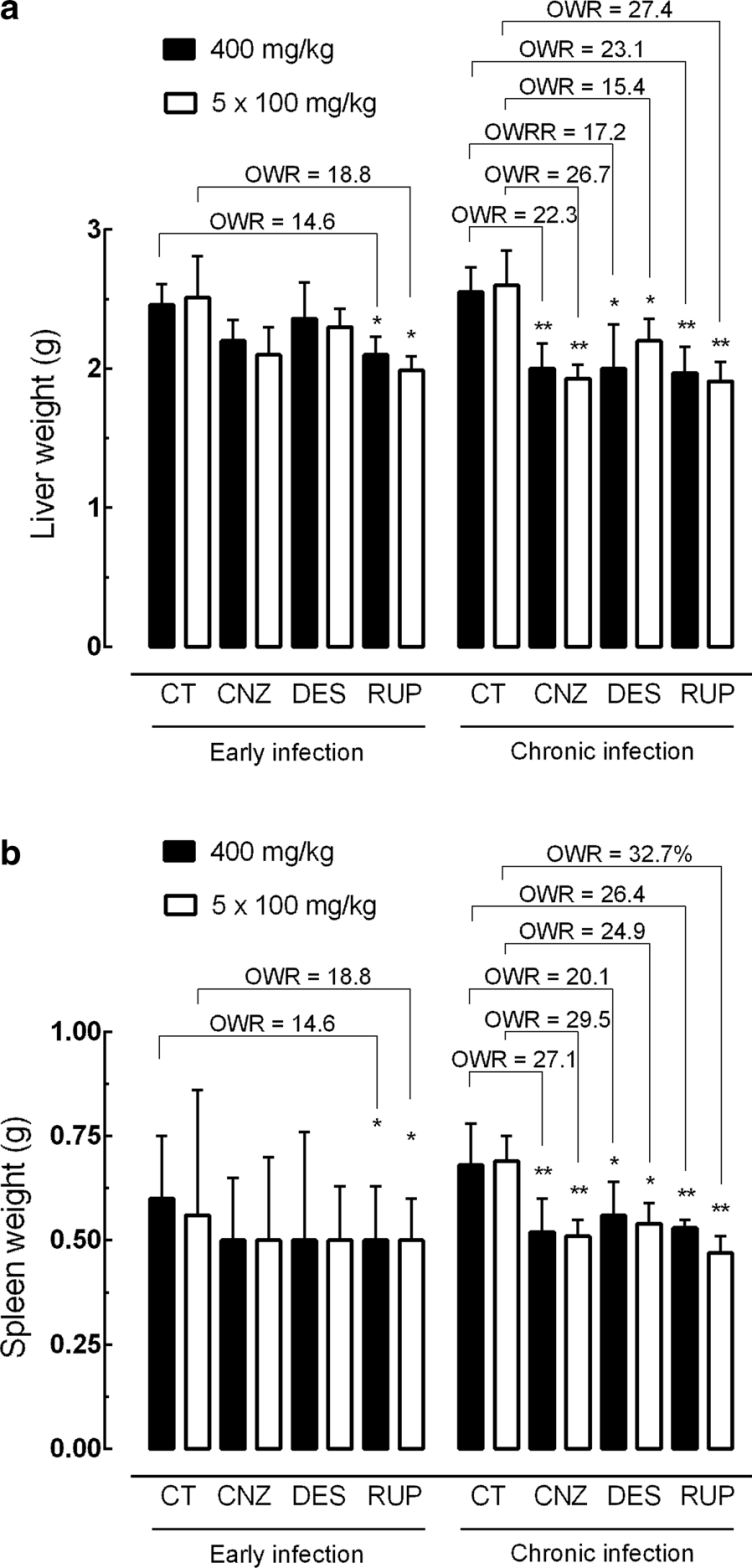
Effect of H1-antihistamine drugs on the organ pathology in a preclinical mouse model. **a** Liver weight. **b** Spleen weight. Drugs were administered orally using a single dose of 400 mg/kg or 100 mg/kg for five consecutive days to mice harboring either early or chronic *S. mansoni* infection. On day 56 post-infection, all animals were humanely euthanized and organ pathology was determined by liver and spleen weights. Data are presented as the mean ± SD (*n* = 5 per group). The numbers represent the percentages of egg reduction *vs* infected untreated control. *P   < 0.05, **P   < 0.01, ***P   < 0.001 compared with infected untreated control groups by Dunnett’s test. *Abbreviations*: OWR, organ weight reduction; CNZ, cinnarizine; DES, desloratadine; RUP, rupatadine

Treatment of *S. mansoni*-infected animals with antihistamines also achieved a significant reduction of hepato- and-splenomegaly, as measured by weight, compared to control infected rodents (Fig. 7). In a chronic infection model, cinnarizine or rupatadine reduced liver mass by 22.3% (ANOVA: *F*_(4, 7)_ = 7.16, *P* = 0.039) to 27.4% (ANOVA: *F*_(4, 7)_ = 7.16, *P* = 0.034) (Fig. 7a) and spleen mass by 26.4% (ANOVA: *F*_(3, 9)_ = 7.94, *P* = 0.038) to 32.7% (ANOVA: *F*_(3, 9)_ = 7.94, *P* = 0.012) (Fig. 7b), whereas a moderate but significant reduction in the liver by 13.6% (ANOVA: *F*_(4, 7)_ = 7.16, *P* = 0.0086) to 18.5% (ANOVA: *F*_(4, 7)_ = 7.16, *P* = 0.034) and spleen by 20.1% (ANOVA: *F*_(3, 9)_ = 7.94, *P* = 0.0041) to 24.9% (ANOVA: *F*_(3, 9)_ = 7.94, *P* = 0.0006) was observed with desloratadine. On the other hand, hepatomegaly and splenomegaly were reduced more slightly in early schistosome infection.

## Discussion

Parasitic flatworm infections are treated by a limited number of drugs and, in most cases, control is reliant upon praziquantel monotherapy. However, praziquantel’s lack of efficacy against immature worms and the emergence of resistance against praziquantel cast a shadow on the global effort to control helminthiasis, as both treatment and control rely significantly on this drug. Since new drugs take a decade or longer to develop, and cost millions of dollars, drug repurposing is a promising approach. Phenotypic screening has successfully identified praziquantel and other anthelmintic agents (e.g. ivermectin and albendazole) that are in veterinary and medical use [31]. In this study, from a screening of 21 H1-antihistamines, we found four drugs which affect the viability of *S. mansoni*.

*In vitro* results showed that two first-generation antihistamines (cinnarizine and promethazine) and two second-generation antihistamines (desloratadine and rupatadine) are highly active against adult schistosomes, with LC_50_ values of 5.8–15.4 µM, whereas the other antihistamines were found to be inactive when screened at 50 μM. Although less potent than praziquantel, which had an LC_50_ value of approximately 0.1 µM [32, 33], cinnarizine, promethazine, desloratadine and rupatadine are more potent than most antischistosomal compounds described so far (for review see [10, 34]). *In vitro* results of this study with cinnarizine are inconsistent with previously reported results, which showed the lack of *in vitro* anti-parasitic activity against the larval [35] and adult [36] stages of *S. mansoni*. Interestingly, assessing the activity profile of an FDA-approved compound library against *S. mansoni* [37], tested promethazine and cinnarizine against adult parasites even at a high concentration of 33 µM and did not see any antischistosomal activity. These inconsistencies are likely a combination of differences in drug concentrations and life stages tested. In addition, it may also be possible that strain differences result in differing drug susceptibilities. For example, Sarhan et al. [36] and Panic et al. [37] used an Egyptian and Liberian strain, respectively, whereas we used a Brazilian strain.

Histamine has an important role as a chemical messenger in physiological responses, neurotransmission, allergic inflammation, and immunomodulation. Its receptors (named H1, H2, H3 and H4) are traditional GPCRs of extensive therapeutic interest [30, 38]. As the target of 33% of all small-molecule drugs, GPCRs are an important class of proteins in drug discovery [39]. Although GPCRs have been described in schistosomes [14, 18], the exact mechanism by which desloratadine, rupatadine, cinnarizine, and promethazine exert their anthelmintic action on schistosomes is still not clear. From a structural point of view, desloratadine, rupatadine and loratadine are similar, but loratadine was inactive *in vitro* against adult schistosomes. Rupatadine contains a 5-methylpyridin-3-yl group connected through a methylene to the basic amine of desloratadine. Interestingly, all four active H1-antihistamines had marked effects on the tegument of *S. mansoni*. However, it is not possible to distinguish causative from consequent action with regard to tegument damage; the drugs may induce it as part of their mechanism of action, or it may be a consequence of parasite death from another mechanism. Unlike nematodes, which are protected by a cuticle, *Schistosoma* species are covered by a living syncytium, called the tegument. This tissue is bounded at its basal surface by a usual invaginated plasma membrane, whereas its apical surface has an atypical heptalaminate appearance [40]. This heptalamellar layer forms many surface pits that substantially enlarge the surface area of the schistosomes. Antihistamines are heterogeneous groups of compounds, with markedly different chemical structures. Comparing the physicochemical properties, the active H1-antihistamines have lower values of TPSA; this membrane permeability parameter may be important to facilitate the permeation of the drugs through the parasite’s tegument and, consequently, the interaction with their molecular target(s). Furthermore, cinnarizine is also a calcium channel blocker, and the possibility of action on the helminth’s calcium channels cannot be excluded. Further studies are needed to elucidate the mechanism of action of the H1-antihistamines in schistosomes.

Cinnarizine, desloratadine, and rupatadine were evaluated in both early and chronic *S. mansoni* infection models in mice. The oral doses of H1-antihistamines that were chosen (single dose of 400 mg/kg and 100 mg/kg daily for 5 days) followed the protocol recommended for a mouse model of schistosomiasis (e.g. [24, 41]). In addition, these drug regimens (single dose or daily, once a day) are in tune with those recommended for the treatment of allergic symptoms. Of note, most H1-antihistamines have an extended duration of clinical activity which allows once‐daily administration. In this study, the treatment with any of the three H1-antihistamines, mainly using 100 mg/kg daily, revealed significant worm burden reductions in animals harboring either chronic or early *S. mansoni* infection. It should be noted that praziquantel treatment exerts high cure rates of 70–90% [42], but it is concerning that some infections in humans and in various other species of animals appear to be refractory to treatment [43, 44]. Importantly, praziquantel has low efficacy against immature parasites (early infection) [45]. Comparatively, oral treatment with cinnarizine, desloratadine, or rupatadine is more effective in early infection than praziquantel. In contrast, praziquantel is more effective in chronic infection that the three H1-antihistamines. Collectively, this finding highlights the advantage of using cinnarizine, rupatadine and desloratadine instead of praziquantel in immature schistosome stages. *In vivo* results of this work with cinnarizine in part mirrored the *in vivo* studies mentioned earlier [36], in that cinnarizine was effective in reducing the worm burden in early infection, surpassing praziquantel.

Egg production, a key mechanism for both transmission and pathogenesis, was also markedly inhibited by antihistamines, and a mitigation effect on hepatomegaly and splenomegaly was also recorded. This result could be attributed to a decrease in the number of parasites as a result of treatment with antihistamines and/or reduction of egg-laying by female helminths. A significant decrease in egg-laying in the intestine or feces has been recently reported with other anthelmintic agents [17, 29, 46]. Moreover, the pathology normally associated with the parasite eggs in the liver and spleen was ameliorated, mainly when antihistamines were given for five days. This finding could be assigned to a decrease in the number of worms and egg-laying. Additionally, it is well established that, in addition to their effects on H1 receptors, antihistamines also possess anti-inflammatory properties and, thus, H1-antihistamine therapy may have contributed to reducing hepatomegaly and splenomegaly in *S. mansoni*-infected mice.

In tandem, rupatadine, cinnarizine, and deslotaradine revealed a moderate reduction in worm and egg burden in mice harboring either early or chronic *S. mansoni* infection. Clinically, a typical dose of these H1-antihistamines is a single 5–20 mg tablet. Even allowing for pharmacokinetic (PK) differences between mice and humans (see dose translation from animal to human studies described by Reagan-Shaw et al. [47]), this dose is much less than 100 mg/kg (let alone 400 mg/kg). Similarly, the maximum serum concentration (Cmax) for these drugs in humans is   < 10 ng/ml (< 0.1 µM), far lower than the concentrations needed to kill schistosomes in culture. Therefore, although these drugs are quite safe, that difference is highly unlikely to support clinical use for schistosomiasis.

## Conclusions

In conclusion, of all the H1-antihistamines tested, promethazine, cinnarizine, desloratadine, and rupatadine are schistosomicidal agents *in vitro*, which is consistent with the extensive structural damage caused by these compounds. In a rodent model of schistosomiasis, desloratadine and mainly rupatadine and cinnarizine greatly reduced worm burden, egg production, and hepatomegaly and splenomegaly. Although the worm and egg burden reductions achieved were all only moderate, comparatively, treatment with any of the three antihistamines is more effective in early infection than praziquantel. On the other hand, the clinical use of H1-antihistamines for the treatment of schistosomiasis is highly unlikely. Finally, the exact mechanism by which these H1-antihistamines exert their anthelmintic effect is still not clear, and further investigation of this property and identification of parasitic-selective ligands that convey this effect are warranted because this could lead to a directed medicinal chemistry effort to identify schistosome-selective compounds.

## Supplementary information

**Additional file 1. Table S1.** Molecular properties of H1-antihistamine drugs.

## Data Availability

The dataset supporting the conclusions of this article is included within the article and its additional file.

## Abbreviations

Cmax: Maximum serum concentration
DMSO: Dimethyl sulfoxide
FBS: Fetal bovine serum
GPCRs: G protein-coupled receptors
LC_50_: 50% Lethal concentration
PK: Pharmacokinetic
SD: Standard deviation
TPSA: Topological polar surface area
