# Supplementary material for: H1-antihistamines as antischistosomal drugs: in vitro and in vivo studies
Source: Parasit Vectors. 2020 Jun 1;13:278. doi: 10.1186/s13071-020-04140-z (PMC7268501; doi:10.1186/s13071-020-04140-z)
Supplement: Supplementary file 1 — Additional file 1. Table S1. Molecular properties of H1-antihistamine drugs. [file 13071_2020_4140_MOESM1_ESM.docx]

**Additional file 1: Table S1*.*** Molecular properties of H1-antihistamine drugs.

| **Drug** | ***S. mansoni* LC_50_ (µM)** | **logS** | **logS @ pH7.4** | **logP** | **logD** | **MW** | **HBD** | **HBA** | **TPSA** | **Flexibility** | **TPSA Heat Map^a^** |
| --- | --- | --- | --- | --- | --- | --- | --- | --- | --- | --- | --- |
| Acrivastine | > 50 | 2.17 | 2.04 | 3.42 | 0.36 | 348.40 | 1 | 4 | 53.43 | 0.21 | 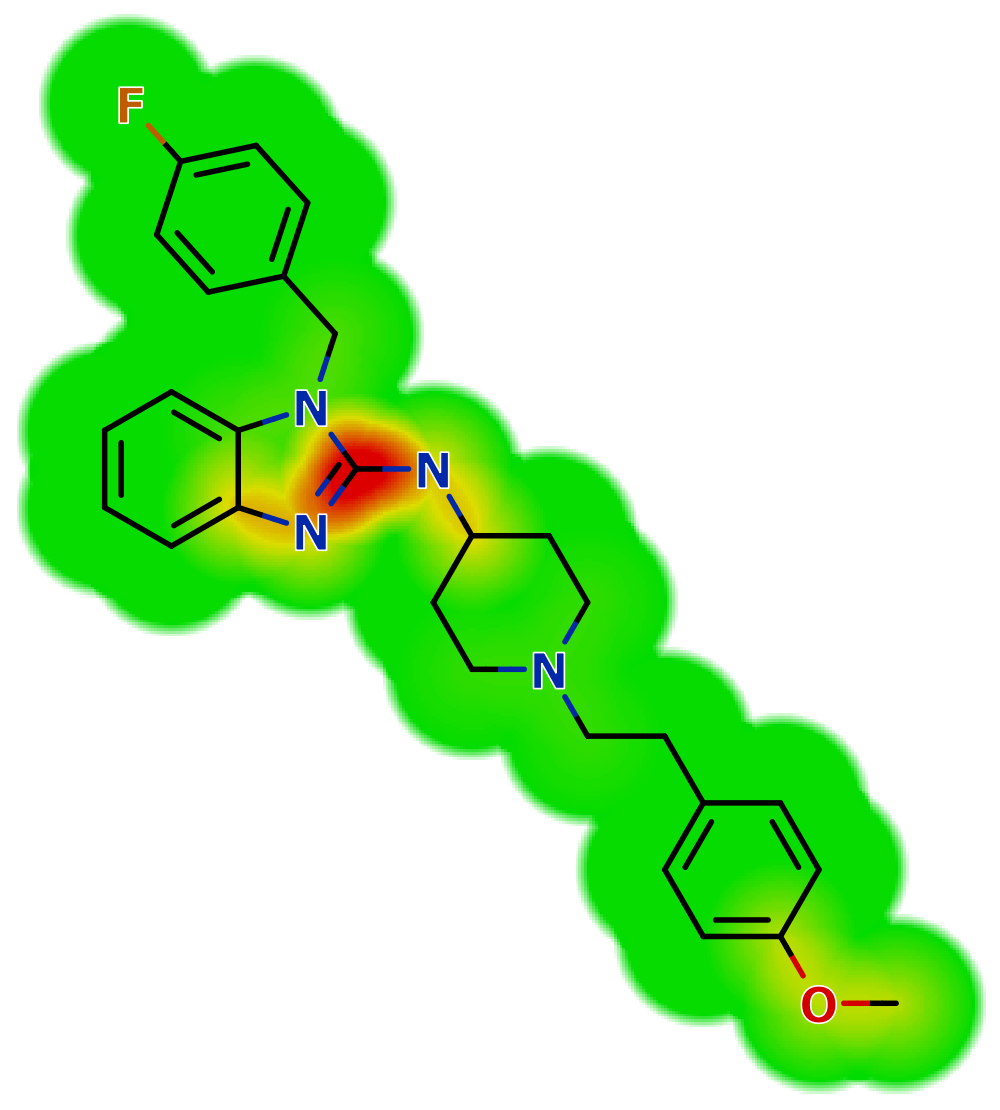 |
| Astemizole | > 50 | 1.13 | 1.62 | 5.70 | 3.78 | 458.60 | 1 | 5 | 42.32 | 0.21 | 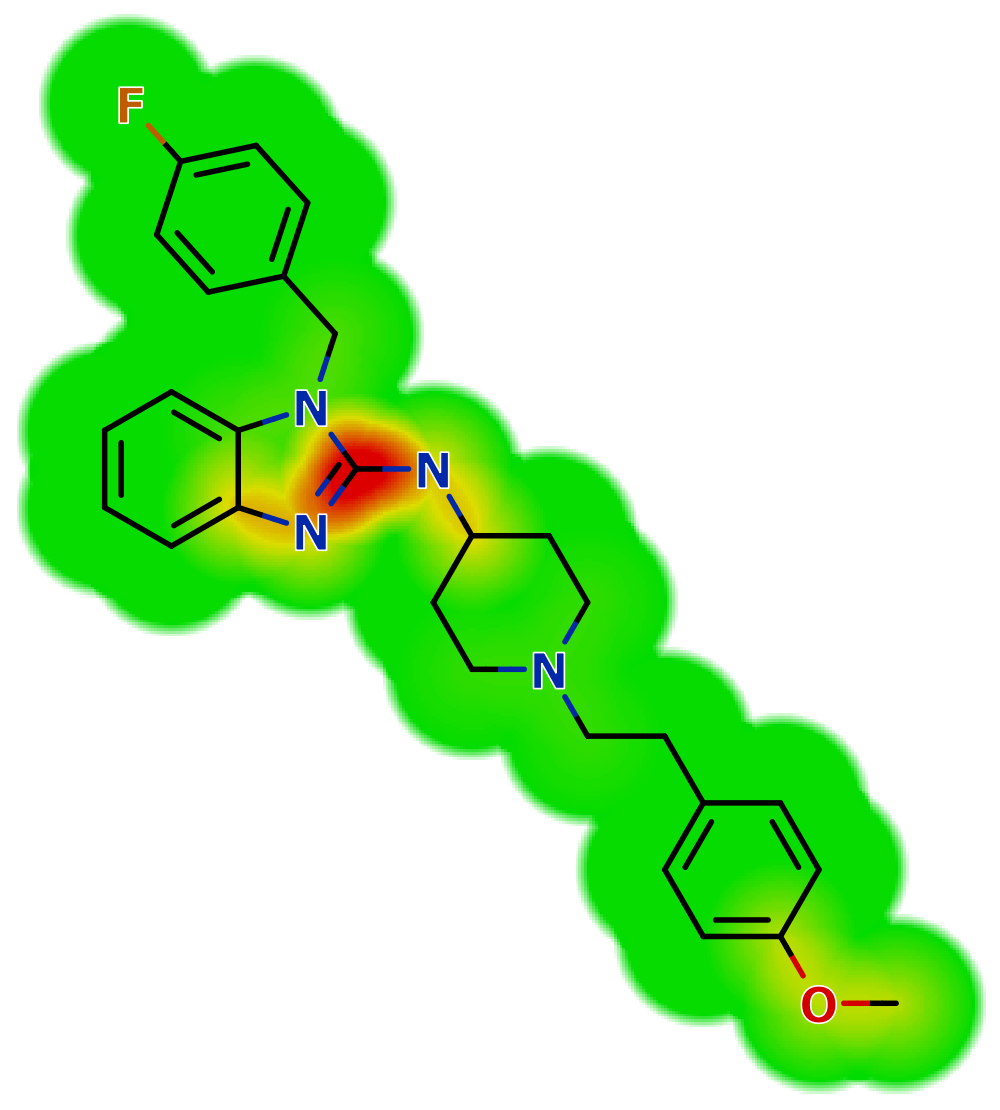 |
| Bilastine | > 50 | 1.68 | 1.32 | 4.96 | 1.35 | 463.60 | 1 | 6 | 67.59 | 0.27 | 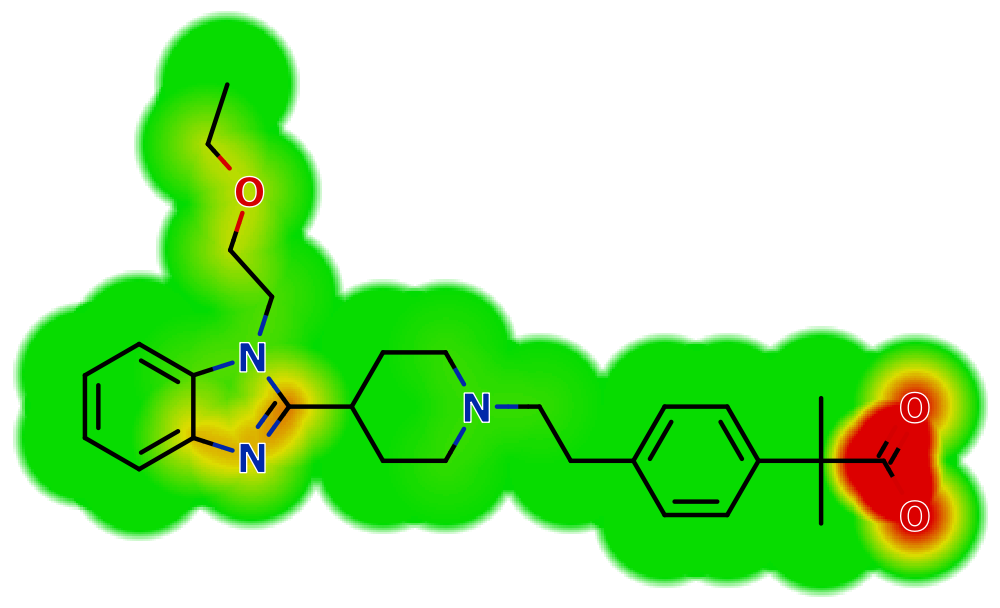 |
| Carebastine | > 50 | 0.23 | 1.27 | 5.48 | 1.92 | 499.60 | 1 | 5 | 66.84 | 0.28 | 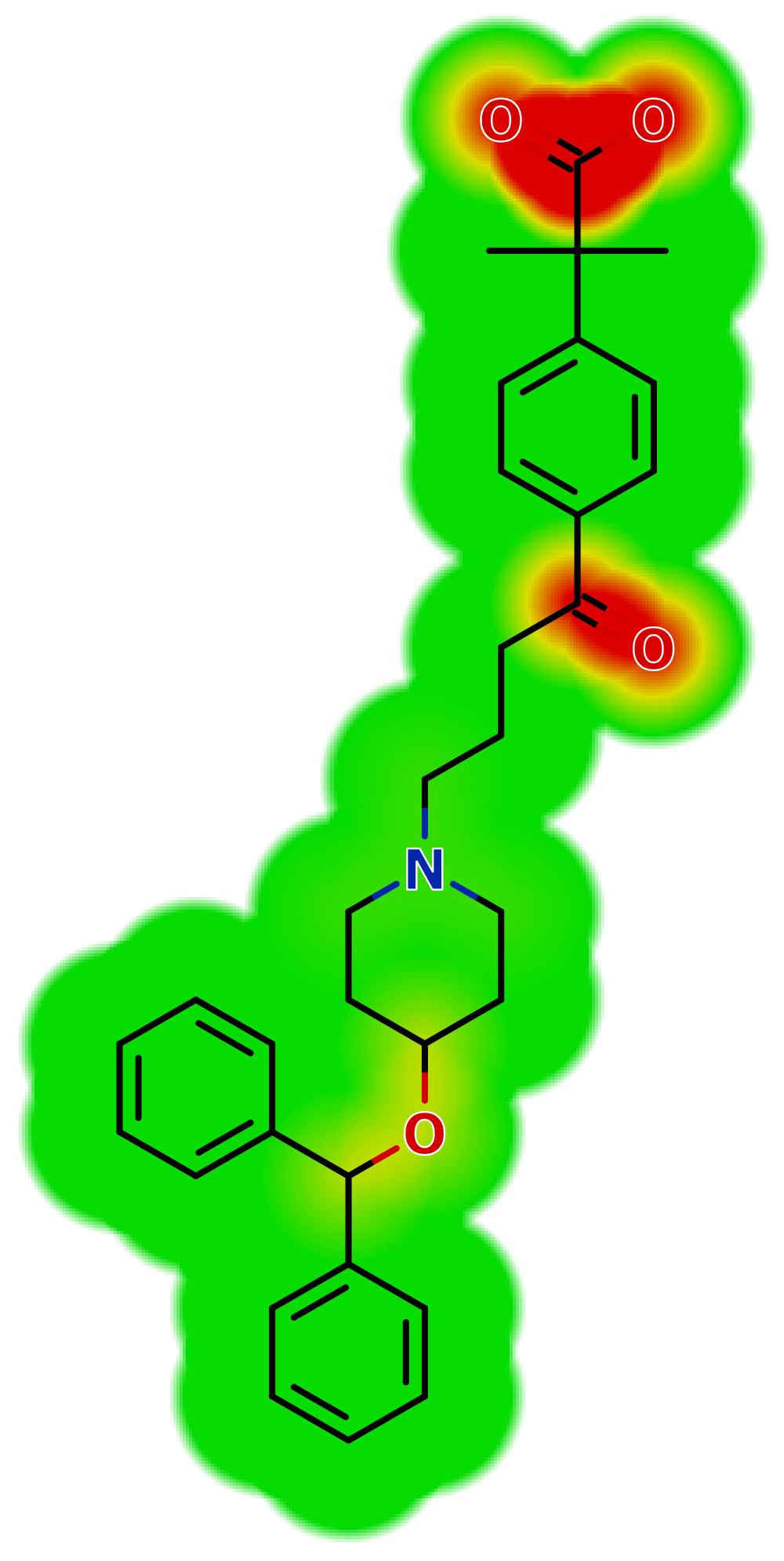 |
| Cetirizine | > 50 | 3.01 | 1.89 | 1.70 | 1.50 | 388.90 | 1 | 5 | 53.01 | 0.28 | 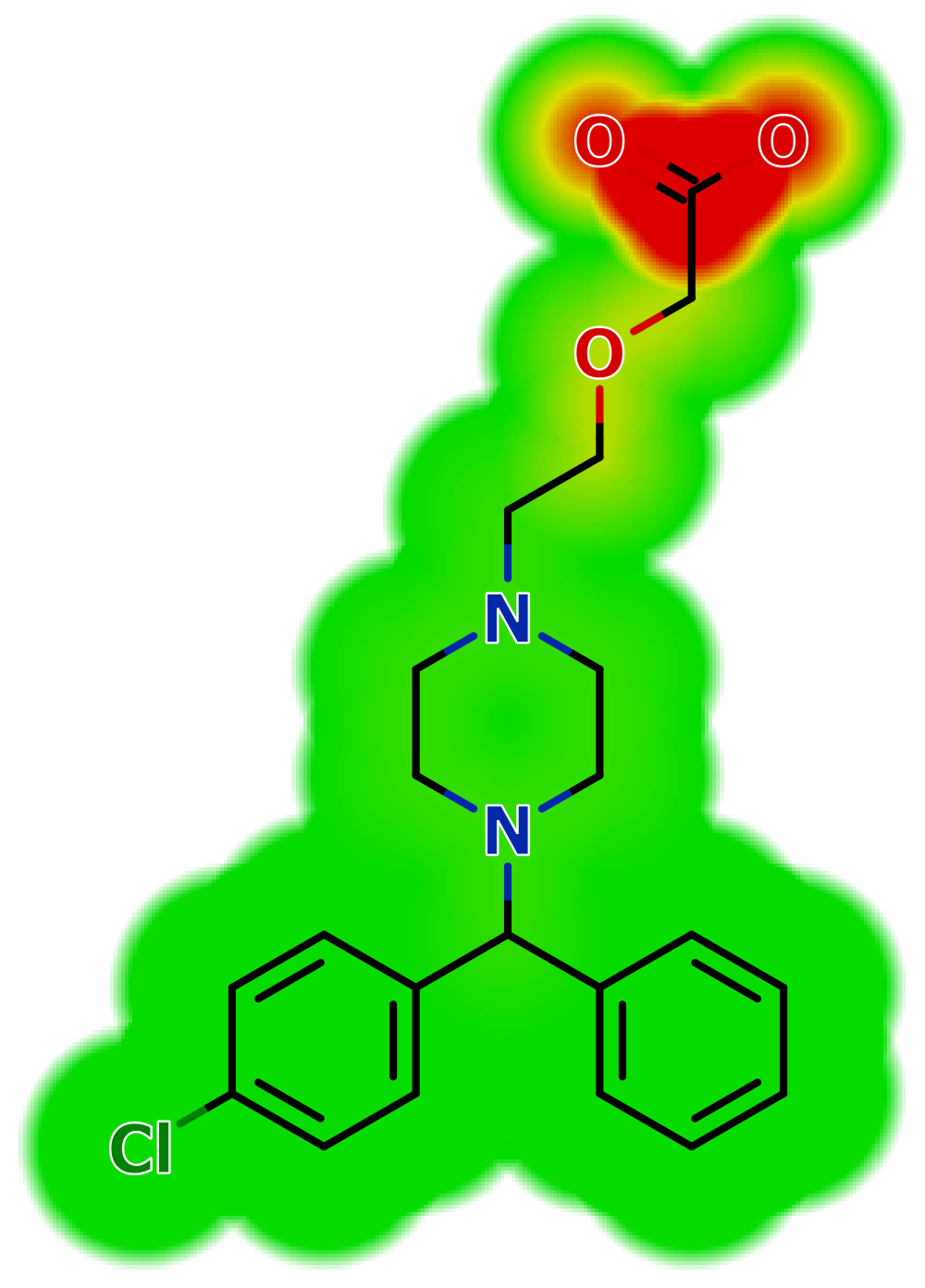 |
| Chlorfeniramine | > 50 | 2.87 | 1.68 | 3.17 | 1.72 | 274.80 | 0 | 2 | 16.13 | 0.25 | 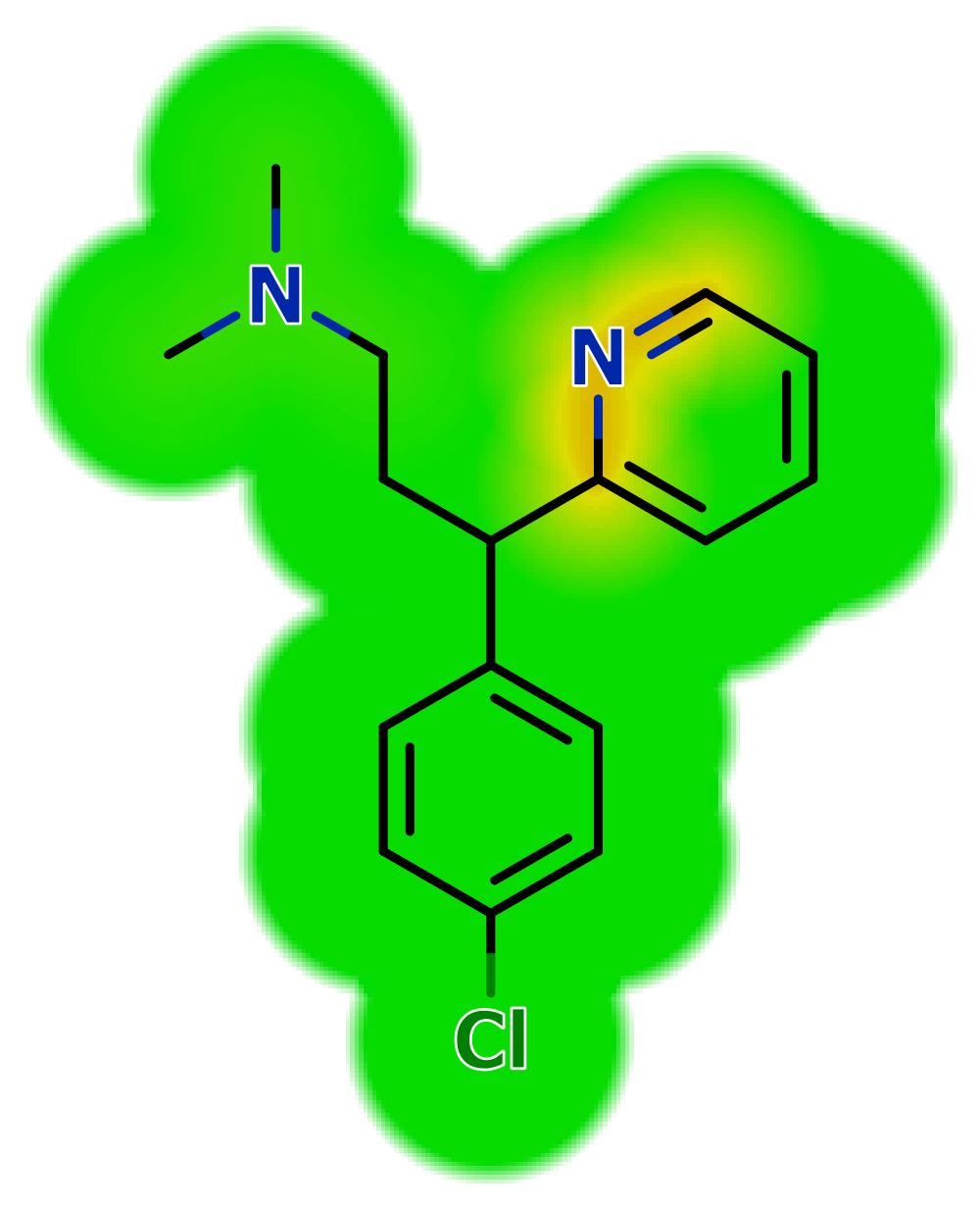 |
| **Cinnarizine** | **7.1** | 2.18 | 1.04 | 5.77 | 3.70 | 368.50 | 0 | 2 | **6.48** | **0.19** | 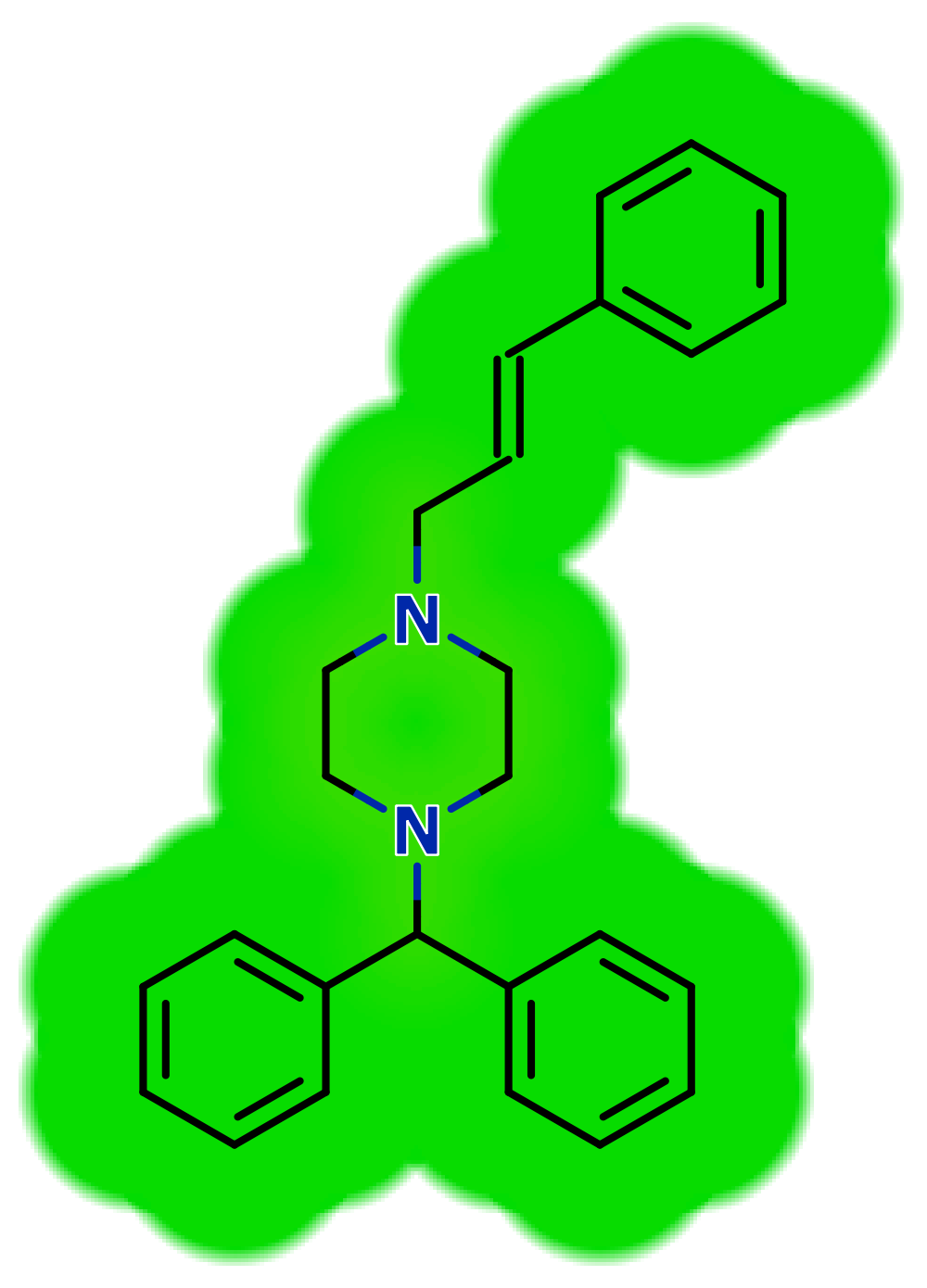 |
| **Desloratadine** | **9.8** | 2.84 | 1.39 | 3.45 | 1.88 | 310.80 | 1 | 2 | **24.92** | **0.00** | 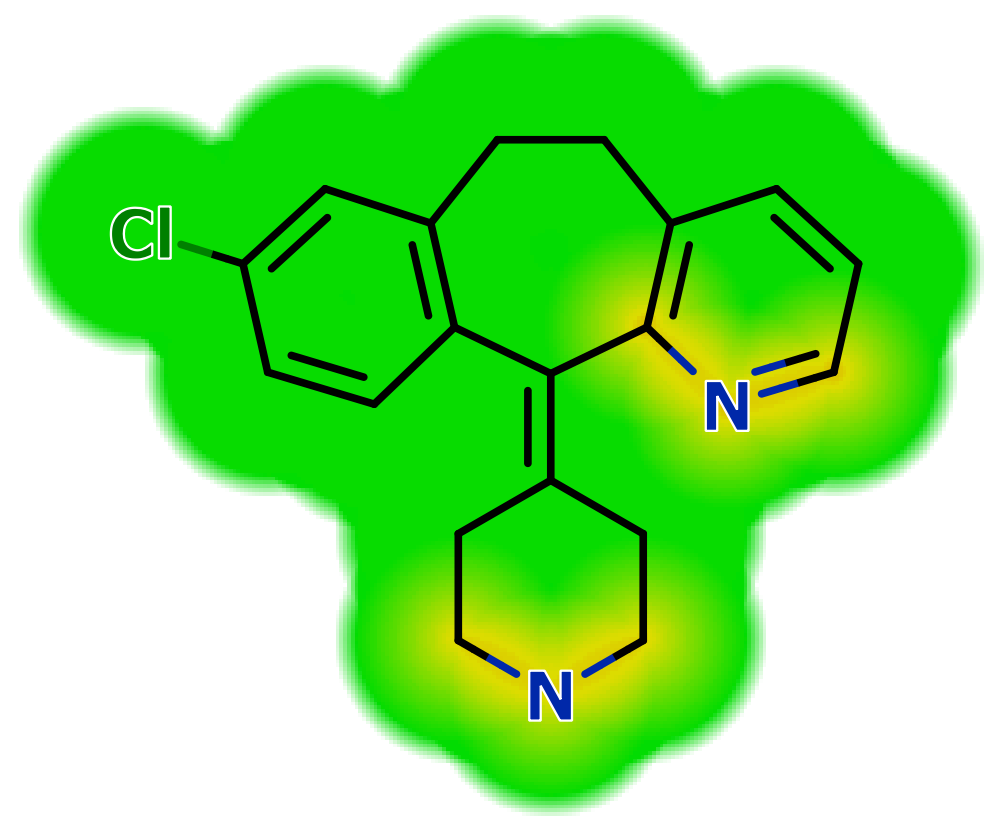 |
| Dexchlorpheniramine | > 50 | 2.87 | 1.68 | 3.17 | 1.69 | 274.80 | 0 | 2 | 16.13 | 0.25 | 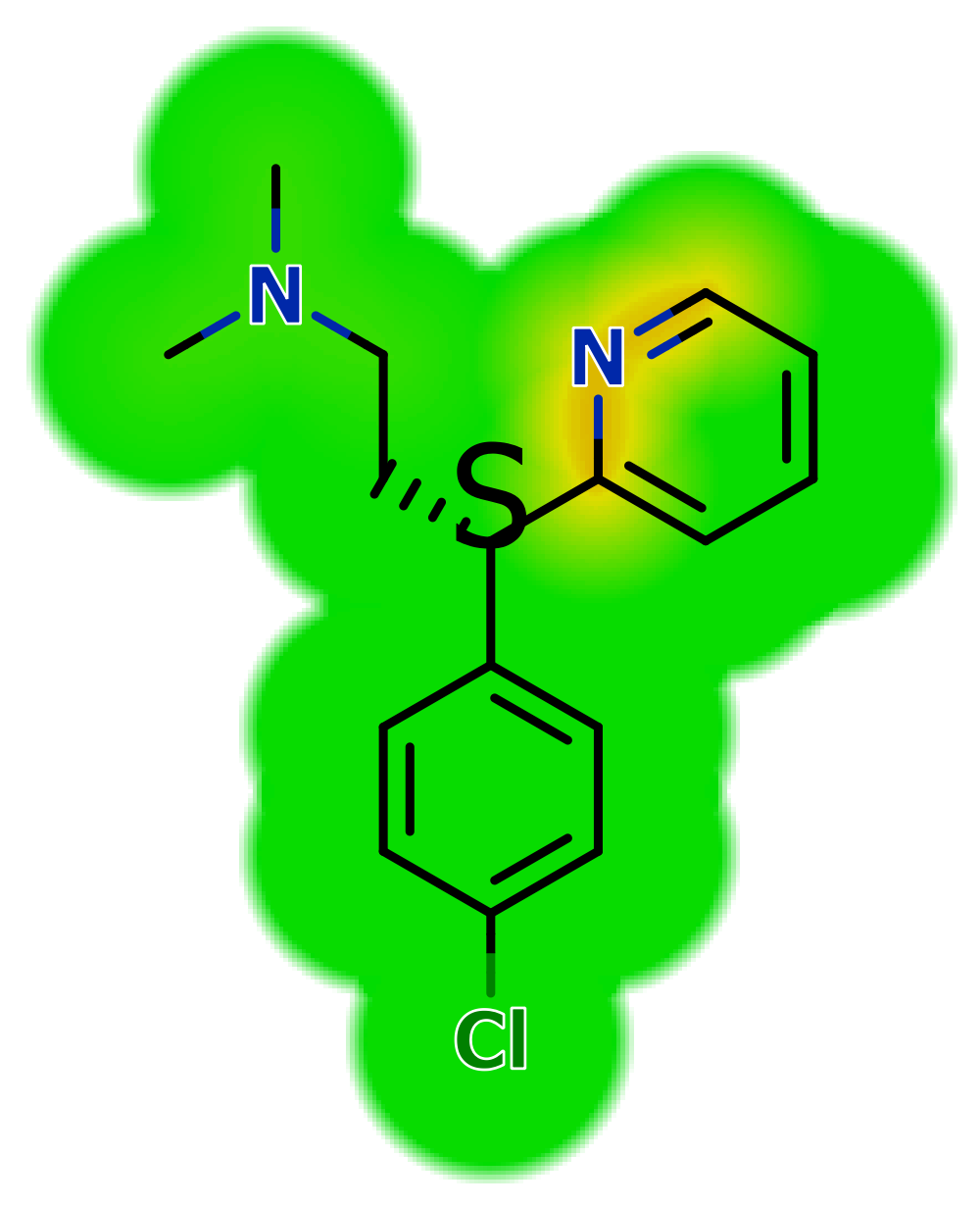 |
| Epinastine | > 50 | 2.47 | 1.42 | 3.51 | -0.06 | 249.30 | 1 | 3 | 41.62 | 0.00 | 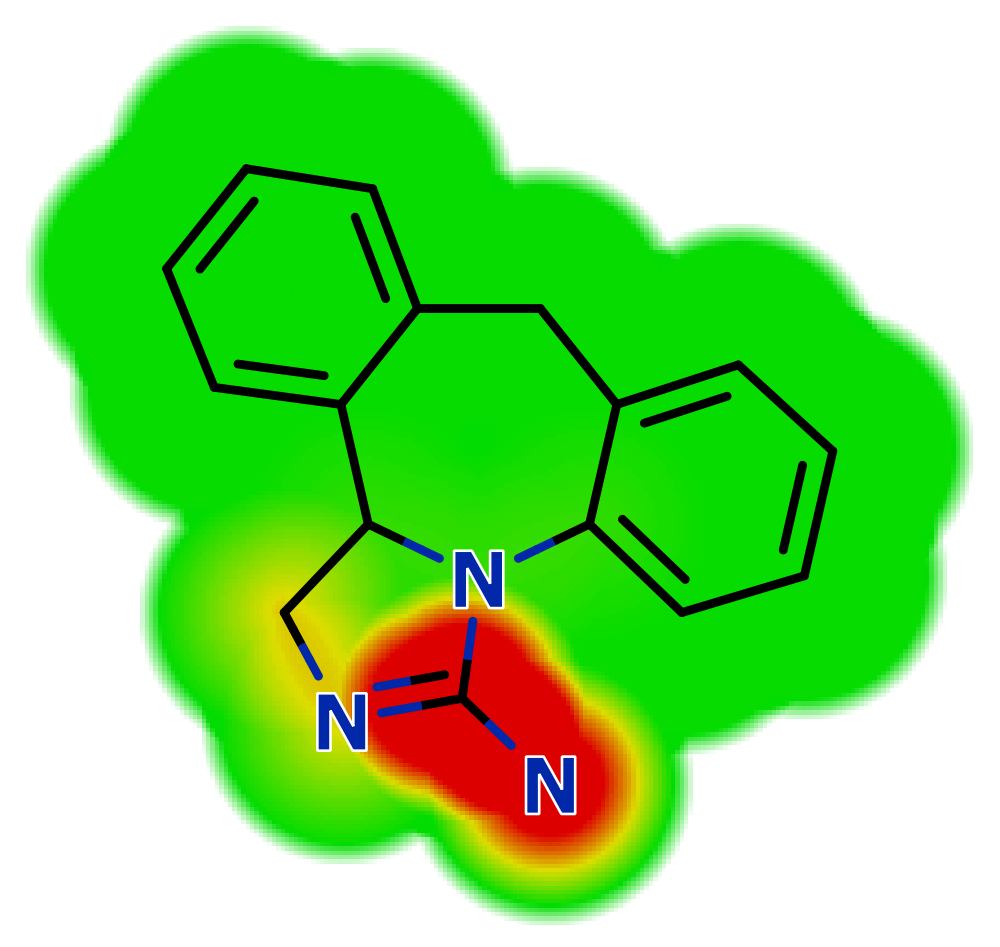 |
| Fexofenadine | > 50 | 1.78 | 1.21 | 4.07 | 1.47 | 501.70 | 3 | 5 | 81.00 | 0.25 | 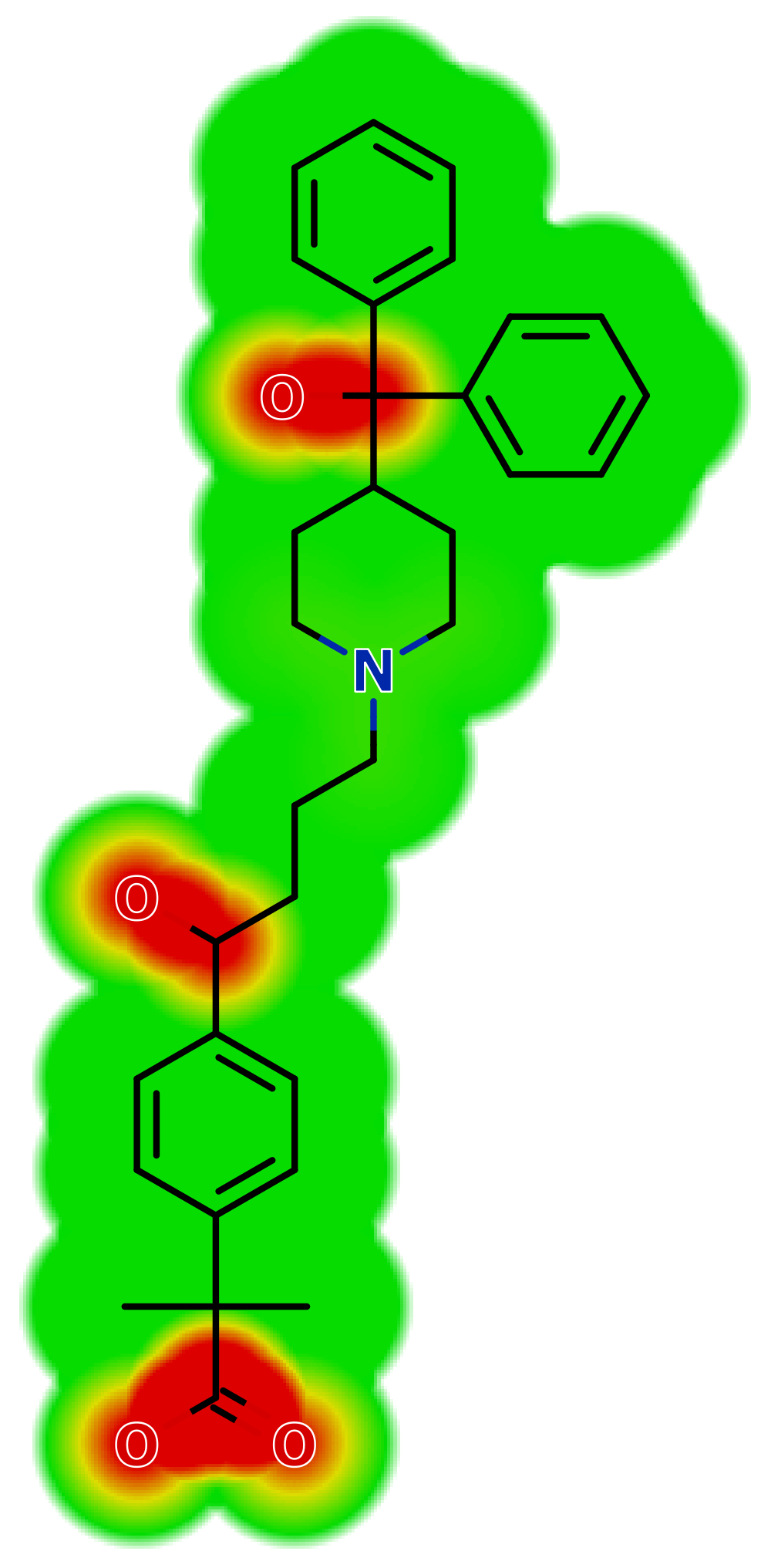 |
| Hydroxyzine | > 50 | 3.31 | 1.82 | 3.25 | 3.10 | 374.90 | 1 | 4 | 35.94 | 0.29 | 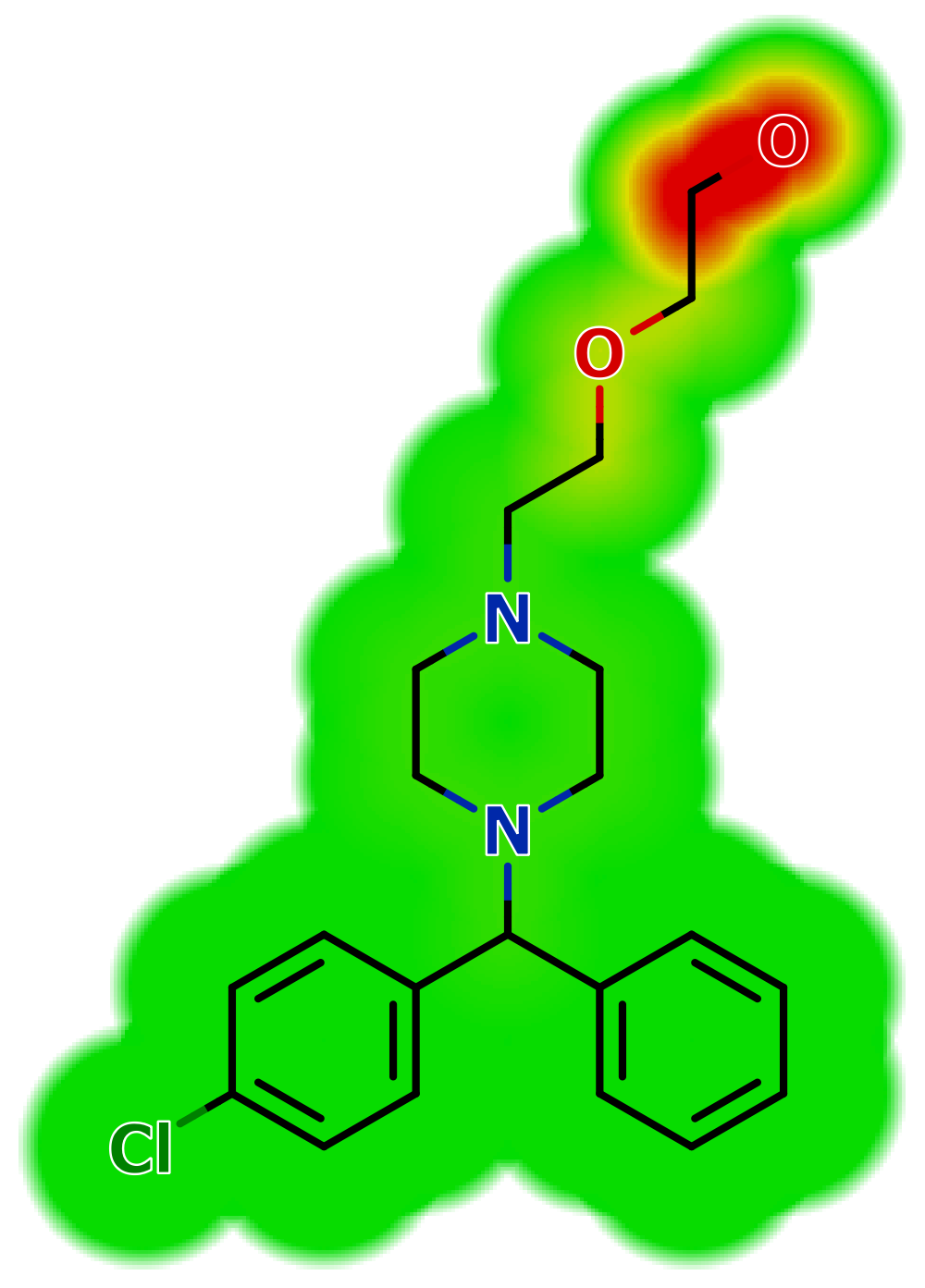 |
| Ketotifen | > 50 | 2.10 | 1.59 | 2.85 | 1.87 | 309.40 | 0 | 2 | 20.31 | 0.00 | 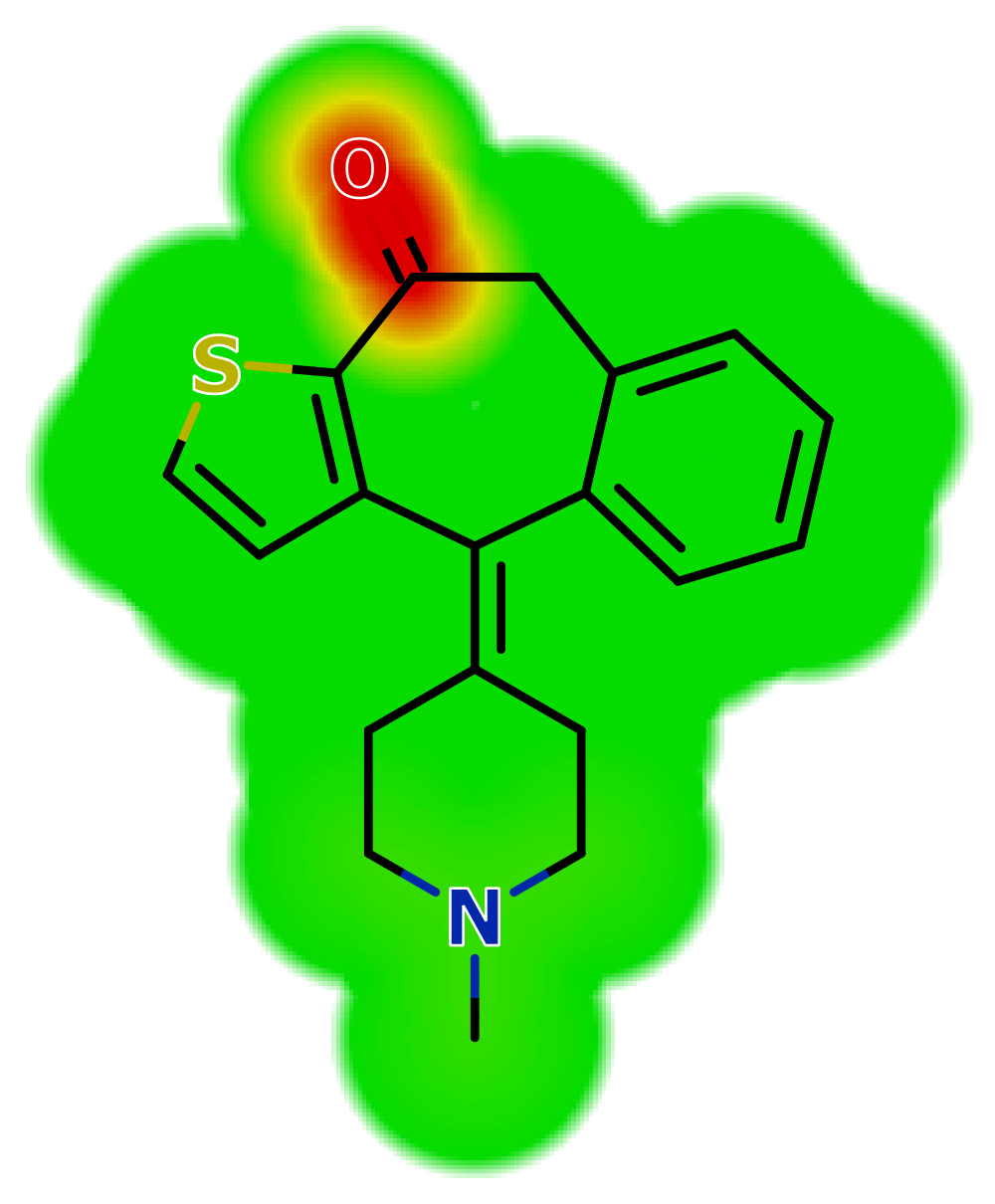 |
| Levocetirizine | > 50 | 3.01 | 1.89 | 1.70 | 1.40 | 388.90 | 1 | 5 | 53.01 | 0.28 | 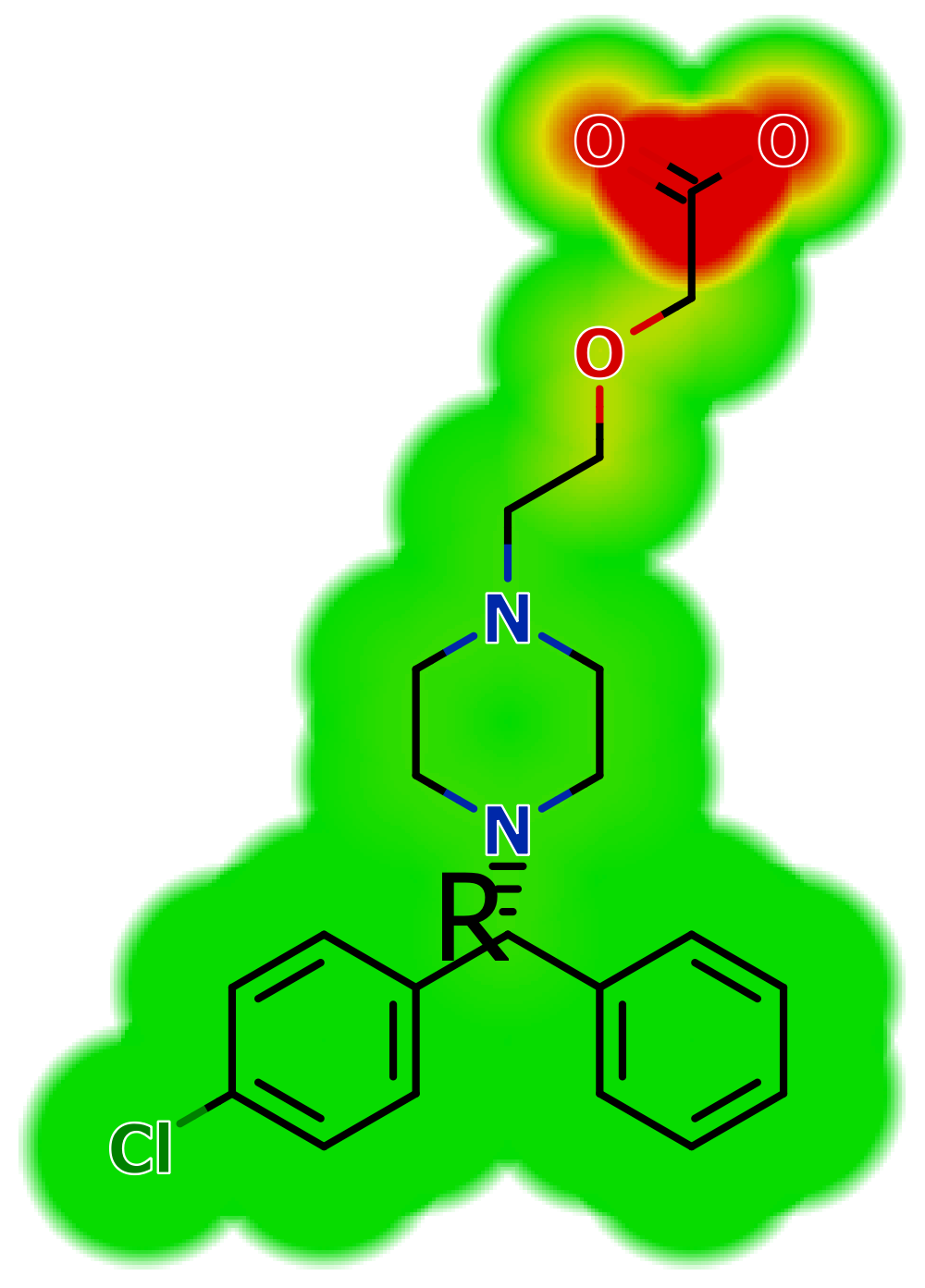 |
| Loratadine | > 50 | 1.29 | 1.29 | 5.20 | 5.20 | 382.90 | 0 | 4 | 42.43 | 0.10 | 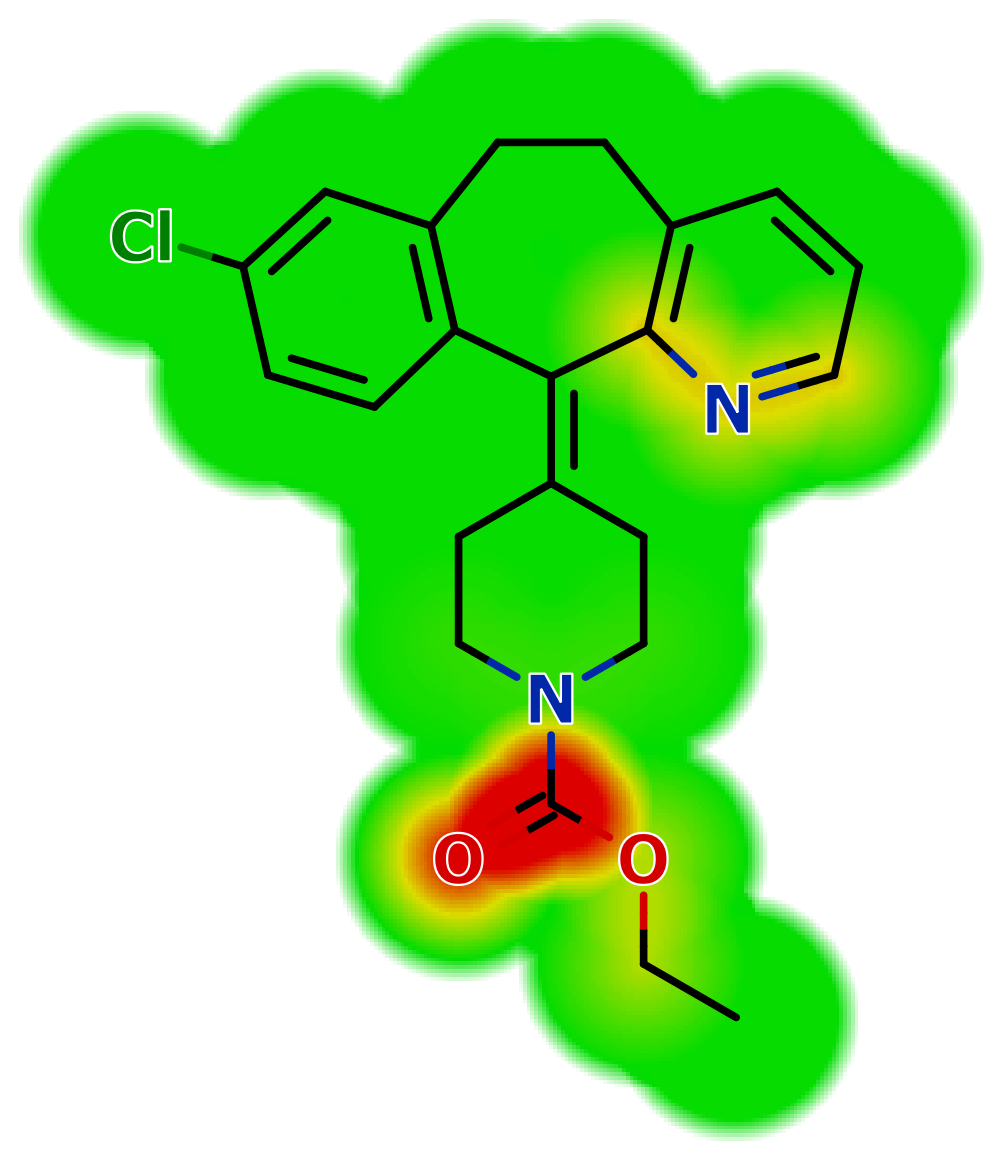 |
| Meclizine | > 50 | 1.74 | 1.13 | 5.76 | 4.34 | 391.00 | 0 | 2 | 6.48 | 0.16 | 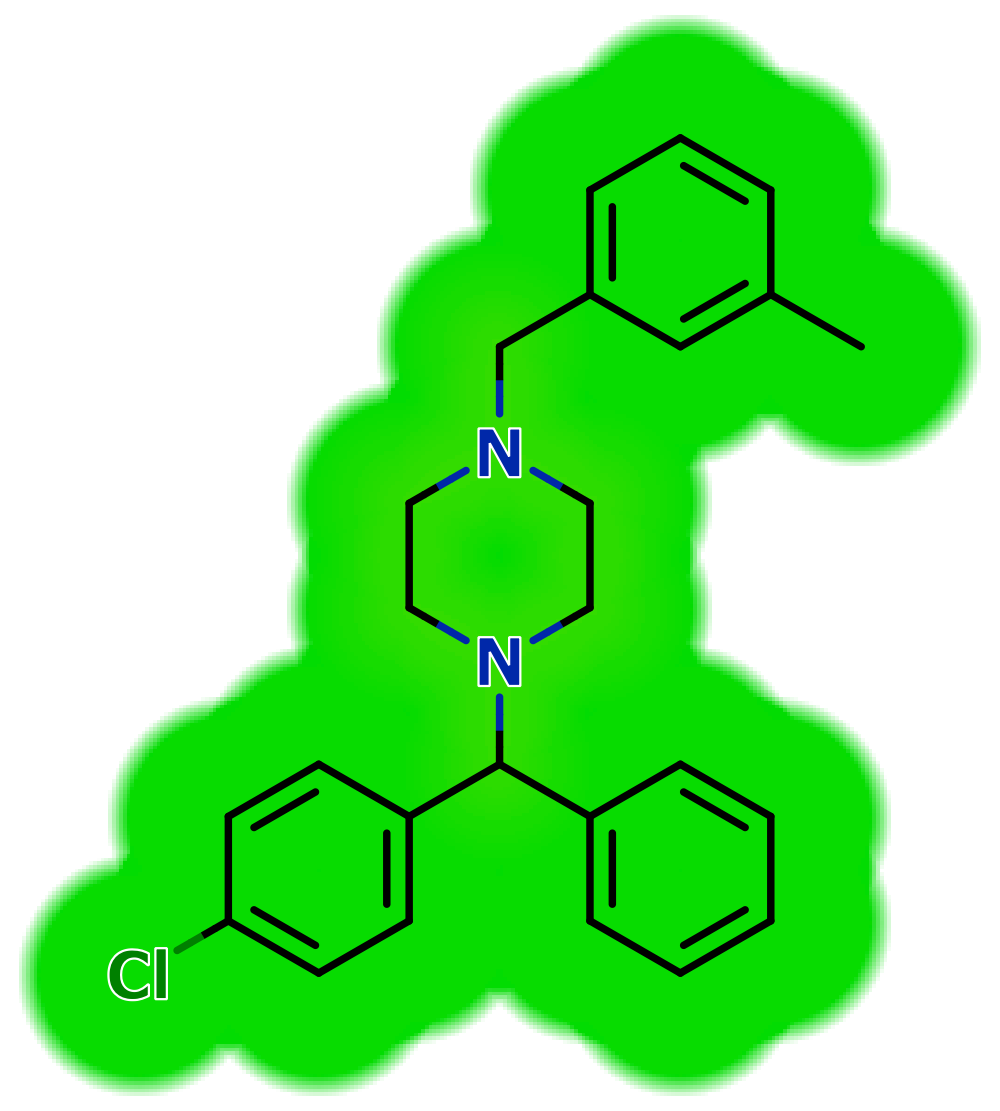 |
| Mizolastine | > 50 | 0.72 | 0.87 | 3.37 | 2.87 | 432.50 | 1 | 7 | 70.05 | 0.14 | 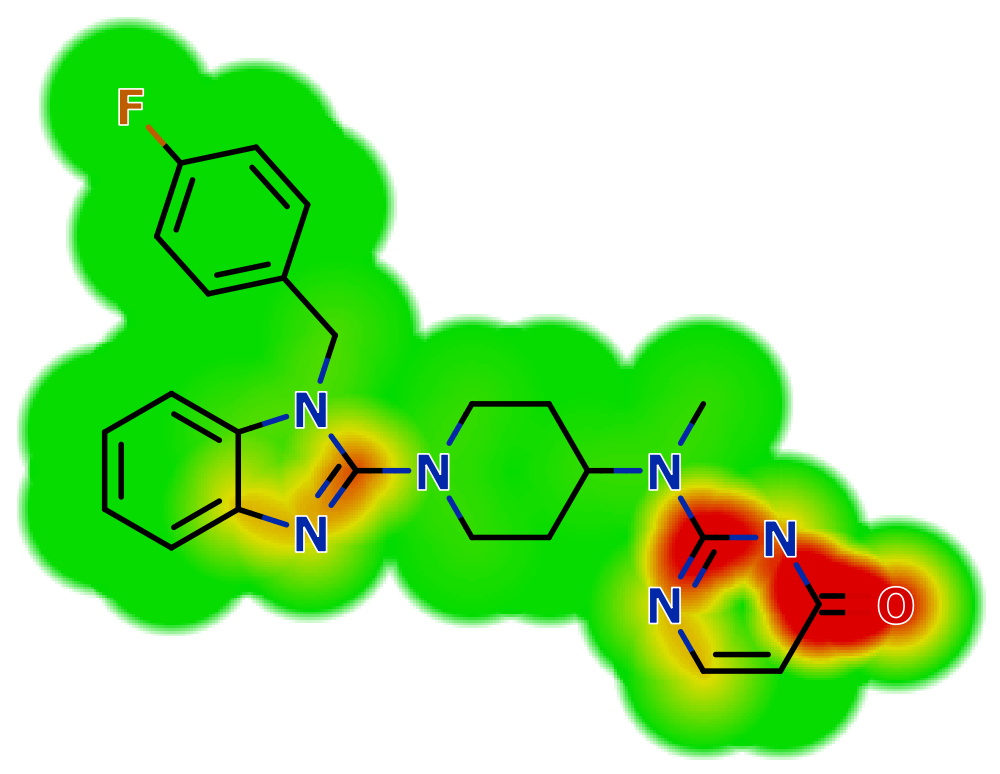 |
| **Promethazine** | **4.9** | 1.74 | 2.18 | 4.81 | 2.52 | 284.40 | 0 | 2 | **6.48** | **0.14** | 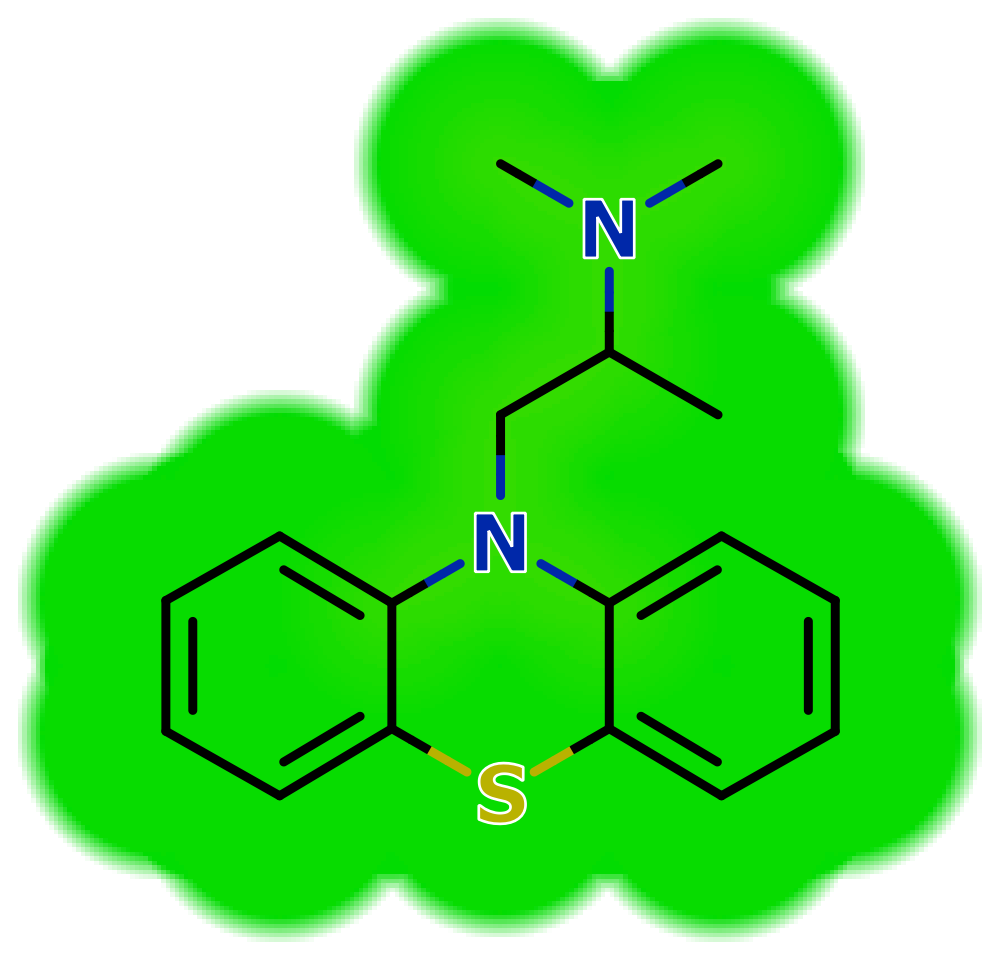 |
| **Rupatadine** | **15.4** | 1.39 | 0.82 | 4.16 | 3.22 | 416.00 | 0 | 3 | **29.02** | **0.06** | 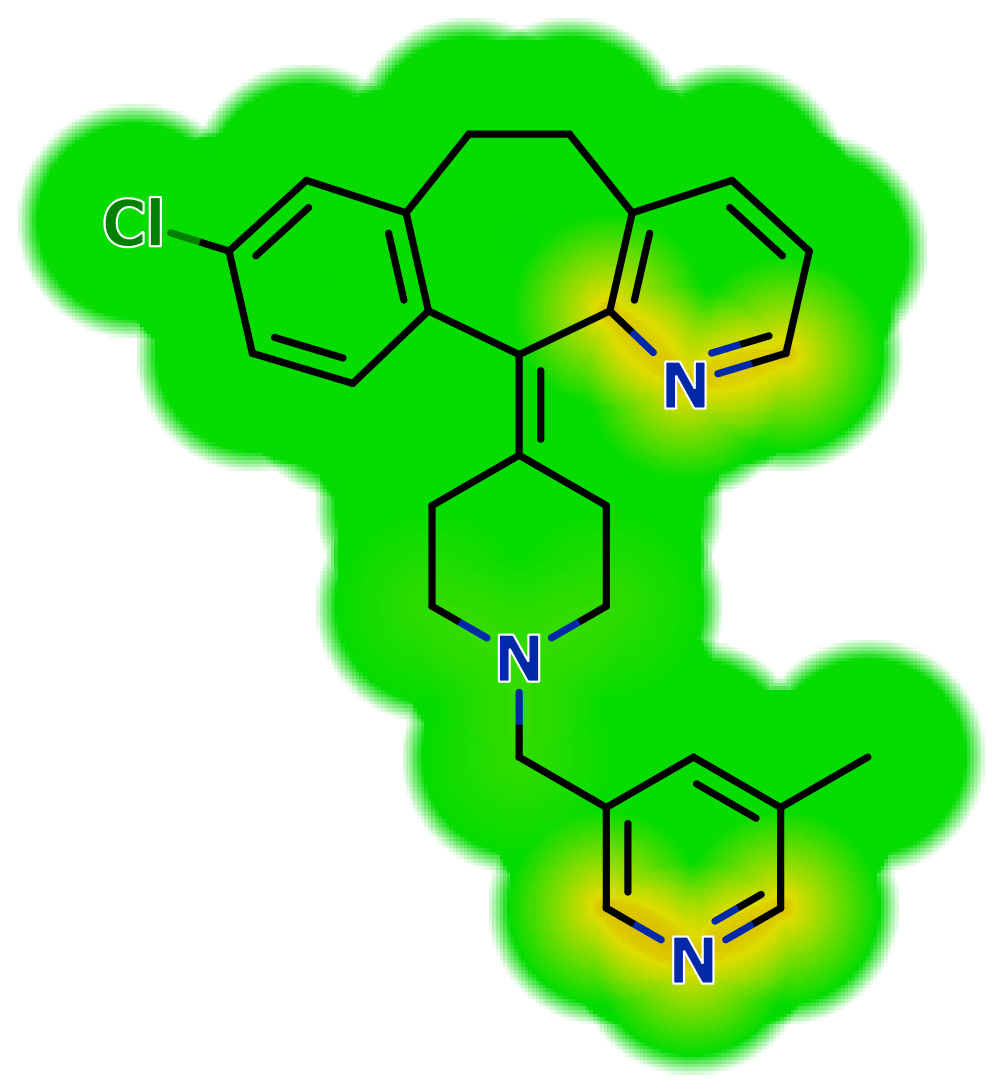 |
| Terfenadine | > 50 | 1.62 | 0.46 | 5.69 | 3.61 | 471.70 | 2 | 3 | 43.70 | 0.24 | 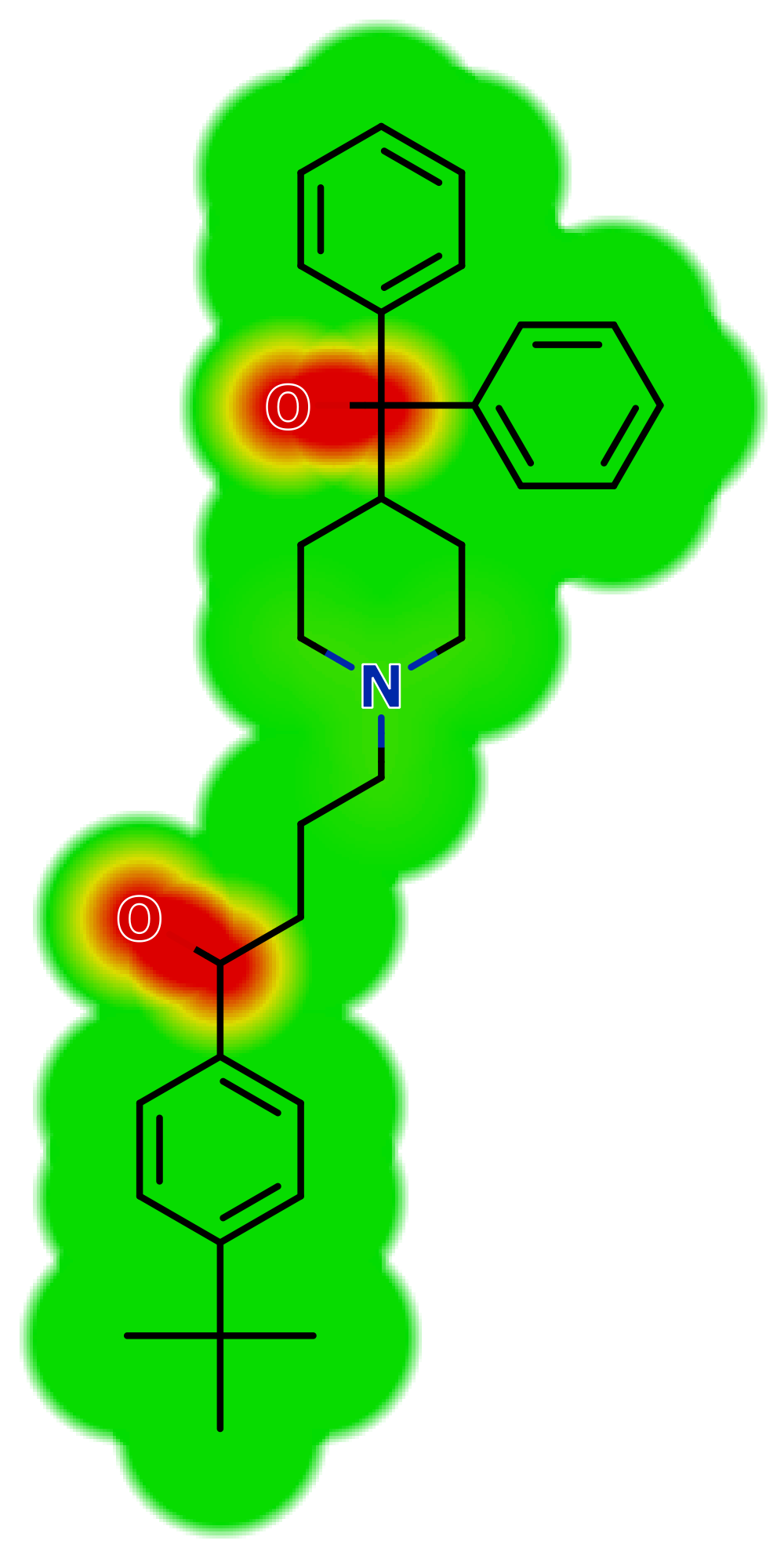 |
| Tripelennamine | > 50 | 3.36 | 1.53 | 3.09 | 1.34 | 255.40 | 0 | 3 | 19.37 | 0.30 | 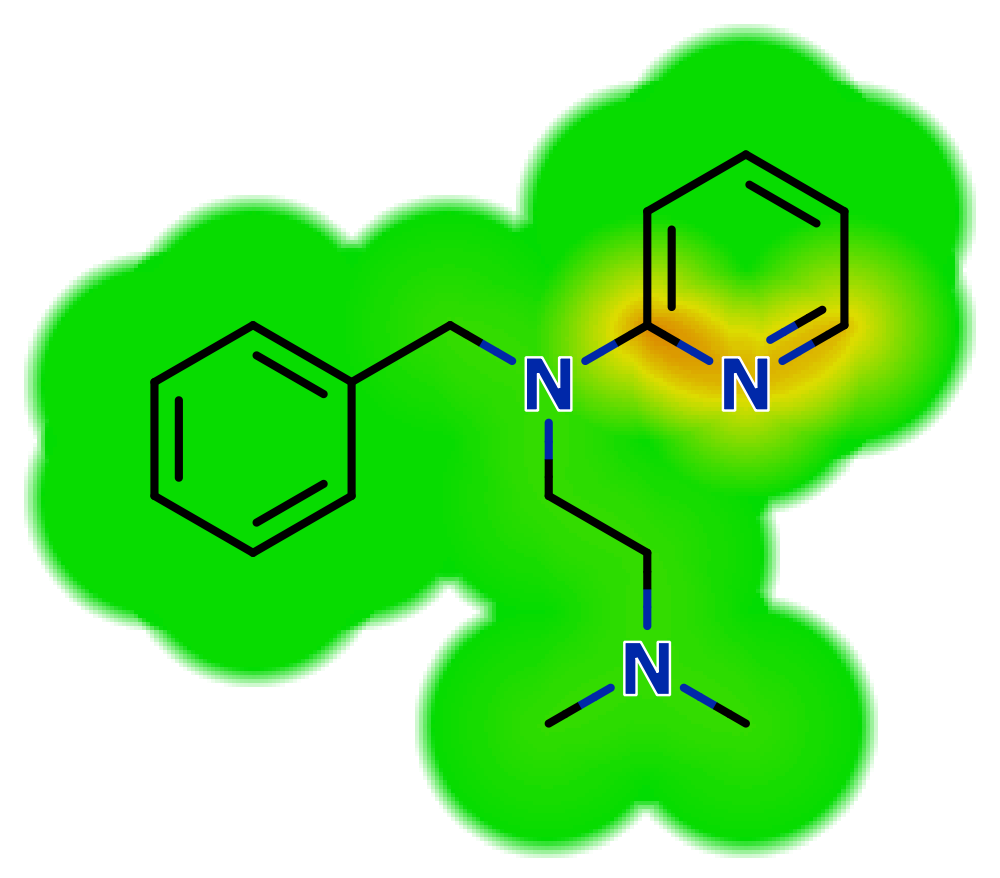 |

^a^ Heat maps for TPSA. The red-yellow regions contribute positively to the property and the green regions have no influence.
